# Novel Mechanisms for IGF-I Regulation by Glucagon in Carp Hepatocytes: Up-Regulation of HNF1α and CREB Expression via Signaling Crosstalk for IGF-I Gene Transcription

**DOI:** 10.3389/fendo.2019.00605

**Published:** 2019-09-03

**Authors:** Jin Bai, Xue Jiang, Mulan He, Ben C. B. Chan, Anderson O. L. Wong

**Affiliations:** School of Biological Sciences, The University of Hong Kong, Hong Kong, China

**Keywords:** IGF-I, glucagon, HNF1α, CREB, signal transduction, gene expression, hepatocytes, grass carp

## Abstract

Glucagon, a key hormone for glucose homeostasis, can exert functional crosstalk with somatotropic axis via modification of IGF-I expression. However, its effect on IGF-I regulation is highly variable in different studies and the mechanisms involved are largely unknown. Using grass carp as a model, the signal transduction and transcriptional mechanisms for IGF-I regulation by glucagon were examined in Cyprinid species. As a first step, the carp HNF1α, a liver-enriched transcription factor, was cloned and confirmed to be a single-copy gene expressed in the liver. In grass carp hepatocytes, glucagon treatment could elevate IGF-I, HNF1α, and CREB mRNA levels, induce CREB phosphorylation, and up-regulate HNF1α and CREB protein expression. The effects on IGF-I, HNF1α, and CREB gene expression were mediated by cAMP/PKA and PLC/IP_3_/PKC pathways with differential coupling with the MAPK and PI3K/Akt cascades. During the process, protein:protein interaction between HNF1α and CREB and recruitment of RNA Pol-II to IGF-I promoter also occurred with a rise in IGF-I primary transcript level. In parallel study to examine grass carp IGF-I promoter activity expressed in αT3 cells, similar pathways for post-receptor signaling were also confirmed in glucagon-induced IGF-I promoter activation and the trans-activating effect by glucagon was mediated by the binding sites for HNF1α and CREB located in the proximal region of IGF-I promoter. Our findings, as a whole, shed light on a previously undescribed mechanism for glucagon-induced IGF-I gene expression by increasing HNF1α and CREB production via functional crosstalk of post-receptor signaling. Probably, by protein:protein interaction between the two transcription factors and subsequent transactivation via their respective cis-acting elements in the IGF-I promoter, IGF-I gene transcription can be initiated by glucagon at the hepatic level.

## Introduction

Glucagon released from the pancreas by functional interaction with insulin is known to play a key role in glucose homeostasis (1), mainly through regulation of gluconeogenesis and glycogenolysis in the liver (2). At the hepatic level, glucagon is also involved in lipid metabolism (3), and recently, a hepato-pancreatic feedback loop has been proposed for regulation of amino acid turnover by glucagon (4). In humans, aberrant expression of glucagon can be associated with pathophysiological or disease conditions (e.g., diabetes, non-alcoholic fatty liver, and glucagon-producing tumor) (5). In mammals, positive correlation between circulating levels of glucagon and growth hormone (GH) (6) and glucagon-induced GH secretion are well-documented (7, 8), suggesting a functional crosstalk of the somatotropic axis with glucagon. However, the effect of glucagon on hepatic expression of IGF-I, the downstream effector for GH, is controversial, as both stimulatory (9) and inhibitory effects (10) have been reported (e.g., in rat hepatocytes). Although the biological actions of glucagon are known to be mediated by the cAMP/PKA and/or PLC/IP_3_/PKC pathways (11, 12), the signal transduction/molecular mechanisms for IGF-I regulation by glucagon are still unknown. In lower vertebrates, including fish species, glucagon, and its receptor are highly conserved in terms of their structure and functions (e.g., glucose production and cAMP coupling) (13, 14). However, little information is available regarding the comparative aspects for IGF-I regulation by glucagon. To date, there is only a single report in salmon hepatocytes showing that glucagon did not alter basal but reduced GH-induced IGF-I mRNA expression at the hepatic level (15). These findings suggest that, similar to mammals, glucagon may also play a role in IGF-I regulation in fish models.

In mammals, IGF-I expression can be activated at the transcriptional level through recruitment of STAT_5_ (16), hepatic nuclear factor 1α (HNF1α) (17), hepatic nuclear factor 3γ (HNF3γ) (18), C/EBP β (19), and p300 to its 5′ promoter (20), which forms a “transcriptional circuitry” mediating GH-induced IGF-I gene expression (21). Among the transcription factors involved in IGF-I promoter activation, the functional role of STAT_5_ and HNF1α appears to be well-conserved in vertebrates and their binding sites can be consistently identified within the promoter of IGF-I gene ranging from fish to mammals (22). In representative species of fish models, e.g., salmon (23, 24) and common carp (25), 5′ deletion of the promoter region with STAT_5_ or HNF1α binding sites (as SBE and HBE, respectively) has been shown to reduce both basal and GH-induced IGF-I promoter activity. However, whether the expression of STAT_5_ and HNF1α can also serve as a target of regulation by hormonal signals to modify IGF-I expression is unclear and remains to be investigated. In our recent study, the 5′ promoter of grass carp IGF-I gene has been cloned. Consistent with the presence of a SBE site in the proximal promoter region, the carp IGF-I promoter was found to be highly responsive to GH activation of JAK_2_/STAT_5_ pathway and this stimulatory effect could be down-regulated by the negative feedback via type II SOCS expression (26). Interestingly, a binding site for cAMP response element binding protein (CREB) (as CRE site) sitting right next to a HBE site was identified downstream of the SBE site in the carp IGF-I promoter and the positions of CRE and HBE sites were also found to be well-conserved in the IGF-I genes of fish species (unpublished results, Wong 2019). This new information based on promoter sequence analysis raises the possibility that CREB may work with HNF1α to trigger IGF-I gene transcription in the carp liver.

Since CREB is the downstream effector of the cAMP/PKA pathway (27, 28) and CREB activation by glucagon has been reported in fish model, e.g., goldfish (29), we hypothesize that HNF1α and CREB may serve as the regulatory targets for glucagon and mediate its effect on IGF-I regulation at the hepatic level in carp species. Grass carp was used as the animal model in our study as (i) it is a representative of Cyprinids with a high market value in Asian countries (~4.6 million tons per year and equivalent to 15.6% of global finfish production; FAO yearbook of Fishery and Aquaculture Statistics 2011), and (ii) bony fish are lower vertebrates leading to the tetrapod lineage and their studies are supposed to provide novel insights on the evolution of endocrine structure/function. Since little/no information is available for HNF1α in carp species, grass carp HNF1α was cloned and characterized for its gene copy number, tissue expression, and bioactivity in stimulating HBE-containing promoter. Using primary culture of grass carp hepatocytes, the functional role of HNF1α and CREB as the targets of regulation mediating the effect of glucagon on IGF-I gene expression was tested in the carp liver. For the signal transduction involved, the post-receptor signaling pathways and their functional interaction at the hepatic level in mediating glucagon regulation of IGF-I, HNF1α, and CREB mRNA expression were examined using a pharmacological approach. Meanwhile, protein:protein interaction between HNF1α and CREB and Pol-II recruitment to IGF-I promoter after glucagon induction were also investigated and correlated with the parallel changes in IGF-I primary transcript levels. The results obtained were further confirmed with transfection studies in αT3 cells to examine IGF-I promoter activation induced by glucagon as well as the functional role of CRE and HBE sites identified in the IGF-I promoter. Our studies for the first time unveil the signal transduction and transcriptional mechanisms for IGF-I regulation by glucagon through up-regulation of HNF1α and CREB expression and transactivation of their respective cis-acting elements in the IGF-I promoter.

## Materials and Methods

### Animals and Test Substances

One-year old grass carp (*Ctenopharyngodon idellus*), were obtained from local wholesale market and maintained in well-aerated water in 250 l aquaria at 20°C under a 12-h light:12-h dark photoperiod for at least 14 days prior to experimentation. During the process of tissue sampling and hepatocyte preparation, the fish was anesthetized in MS222 (0.05%; Sigma, St. Louis, MO) and spinosectomized according to the protocol approved by the Committee for Animal Use at the University of Hong Kong (Hong Kong). Human glucagon and pharmacological agents for signaling targets/kinases, including forskolin, MDL12330A, H89, GF109203X, TPA, PD98059, SCH772984, Ly294002, API-2, rapamycin, N-6-benzoyladenoxine-3′,5′-cAMP (6Bnz-cAMP), 8-(4-chlorophenylthio)-3′,5′-cAMP (8cpt-cAMP), and 2-aminoethoxy-diphenyl borate (2-APB) were acquired from Calbiochem (San Diego, CA), while chelerythrine, 1,2-dioctanoyl-*sn*-glycerol (DiC-8), actinomycin D and cycloheximide were obtained from Sigma and edelfosine and 8-(4-chlorophenylthio)-2′-O-methyladenosine-3′,5′-cAMP (8cpt-2Me-cAMP) were purchased from Tocris (Ellisville, MO). For transfection studies, the expression vector for mouse HNF1α and HNF1α reporter pGL_3_.HNF1α.Luc carrying tandem repeats of HBE sites in its 5′ promoter were procured from Yeasen Biotech (Shanghai, China).

### Molecular Cloning and Characterization of Carp HNF1α

Grass carp HNF1α cDNA was cloned using 5′/3′RACE with primers designed according to the conserved regions of zebrafish HNF1α with total RNA isolated from the carp liver as the template as described previously (26). Sequence alignment, 3D protein modeling and phylogenetic analysis were conducted using Clustal W (http://www.ebi.ac.uk/clustalw), SWISS-MODEL (http://www.expasy.org/swissmod) and MEGA 6.0 (http://www.megasoftware.net/index.html), respectively. To evaluate the gene copy number of HNF1α, Southern blot was performed in DNA sample purified from the whole blood of grass carp according to the standard protocol (30). For tissue expression profiling of HNF1α, RT-PCR was conducted in selected tissues and brain areas using primers specific for HNF1α transcript with parallel PCR of β actin as the internal control. Using LC/MS/MS, protein expression of HNF1α in the carp liver was also examined using a SCIEX TripleTOF-5600 system (AB SCIEX, Concord, ON, Canada) (31). To confirm the functionality of the newly cloned cDNA, the ORF of carp HNF1α was cloned into pcDNA/Zeo to generate the expression vector pcDNA/HNF1α. The vector was then used in promoter study in αT3 cells with co-transfection of the pGL_3_.HNF1α.Luc reporter and TK-Renilla (as internal control) to test the ability of carp HNFα expression in transactivating target promoter with ×4 tandem repeats of HBE sites. In this experiment, parallel expression of mouse HNF1α was used as a positive control.

### IGF-I, CREB, and HNF1α mRNA Measurement in Carp Hepatocytes

Primary culture of grass carp hepatocytes was prepared by collagenase digestion method (26) and maintained in DMEM/F12 medium at a seeding density of ~0.8 × 10^6^ cells/ml/well in PEI-precoated 24-well plates. After drug treatment, total RNA was extracted with removal of genomic DNA and reversely transcribed as described previously (30). The samples prepared were then subjected to real-time PCR for IGF-I, CREB, and HNF1α mRNA measurement using primers and PCR conditions as described in Supplemental Table 1 using a RotorGene-Q System (Qiagen, Hilden, Germany). In our studies, serial dilutions of plasmids with the ORF of target genes were used as the standards for data calibration and parallel measurement of 18S RNA expression was used as the internal control. After the assays, the authenticity of the respective PCR products was routinely confirmed by melting curve analysis.

### Western Blot for CREB and HNF1α in Carp Hepatocytes

To study the effects of glucagon on CREB activation and CREB and HNF1α protein expression in the carp liver, Western blot was performed in carp hepatocytes (26) after glucagon treatment for the duration as indicated. During the process, cell lysate prepared in RIPA buffer with inhibitor cocktails for proteases and phosphatases (Roche) was subjected to SDS-PAGE and transblotted onto Immobilon membrane (Millipore) followed by Western blot with antibodies for phosphorylated CREB (P-CREB, 1:500; Santa Crutz, Dallas, Texas), total CREB (T-CREB, 1:500; Calbiochem) and HNF1α (1:2,500; Abcam, Eugene, OR), respectively. After incubation with HRP-conjugated secondary antibody (1: 5,000, Bio-Rad, Hercules, CA), Western blot signals were visualized using the SuperSignal WestPico Reagent (Pierce, Rockford, IL). Parallel blotting for β actin was also conducted to serve as the internal control using an Actin AB-1 Kit (Calbiochem). To test the effect of glucagon on protein:protein interaction of CREB and HNF1α, cell lysate prepared from hepatocytes with glucagon treatment was subjected to co-immunoprecipitation (32) using a Crosslink Immunoprecipitation Kit (Thermo Scientific, Waltham, MA). Protein pull-down was conducted with HNF1α antibody (1:100) followed by immunoblotting using the antibodies for phosphorylated and total CREB (1:500), respectively, and parallel pull-down with mouse IgG antibody (1:100) was used as a negative control.

### RNA Pol-II ChIP and IGF-I Primary Transcript Measurement

To shed light on the effect of glucagon on IGF-I gene transcription in the carp liver, chromatin immunoprecipitation (ChIP) of IGF-I promoter was performed in carp hepatocytes using an EZ ChIP Assay Kit (Millipore, Before, MA) with the antibody for RNA polymerase II (Pol-II). After exposure to glucagon, hepatocytes were fixed in 1% formaldehyde for 10 min followed by quenching with 1 M glycine for 5 min at room temperature. Following cell harvest with a cell scraper (Thermo Scientific), hepatocytes (~4 × 10^6^ cells) were pelleted by centrifugation, dissolved in 200 μl SDS lysis buffer with protease inhibitor cocktail II (Roche) and sonicated using a M220 Focused-Ultrasonicator (Covaris, Woburn, MA) with peak incident power of 75 W and duty factor at 10% with 200 cycles per burst. A 5 μl aliquot of the cell lysate containing sheared chromatin with fragment size of 0.5–0.8 kb was saved as the input control (1:100 dilution) and the rest of the sample was subjected to immunoprecipitation using the antibody for RNA Pol-II (1:1,000, Millipore) according to the instructions of the assay kit with minor modifications (33). After decrosslinking of the chromatin pulled down by ChIP for 5 h with 65°C heating, the amount of IGF-I promoter pulled down with RNA Pol-II was monitored by real-time PCR using primers covering the proximal region of IGF-I promoter (Supplemental Table 1). In this experiment, parallel ChIP without Pol-II antibody (as a “no-antibody” control) or with the mouse IgG antibody (as the “antibody control”) was used as the negative control. The specificity of PCR signals detected was validated with genomic DNA as a positive control and “Mock ChIP” with Pol-II antibody but no chromatin as a negative control (as “Mock Ctrl”). To confirm that Pol-II association with IGF-I promoter could be correlated to IGF-I gene transcription, time course, and dose dependence studies were also performed in carp hepatocytes to examine the effects of glucagon on IGF-I primary transcript levels with real-time PCR using primers covering the junction between exon III and intron III of carp IGF-I gene (Supplemental Table 1). In our studies, serial dilutions of plasmids carrying the amplicon for IGF-I promoter/primary transcript were used as the standards for data calibration. Real-time PCR of IGF-I promoter in chromatin input was used as the loading control for Pol-II ChIP and parallel measurement of 18S RNA expression was used as the internal control for IGF-I primary transcript.

### IGF-I Promoter Activity Expressed in αT3 Cells

To examine the role of promoter activation in glucagon-induced IGF-I gene transcription, a 1.07 kb 5′ promoter of carp IGF-I gene was subcloned into the luciferase-expressing reporter pGL_3_.Basic (Promega) to generate the pIGF1(-1077).Luc construct for transfection studies in αT3 cells. Based on our validation in nine cell lines, αT3 cells were the only cell line with glucagon inducibility for carp IGF-I promoter and post-receptor signaling comparable with carp hepatocytes. To delineate the promoter region responsible for glucagon induction, 5′ deletion analysis of the IGF-I promoter carried in pIGF1(-1077).Luc was conducted using a Mung Bean Nuclease 5′ Deletion Kit for Kilo-Sequencing (TaKara Bio Inc., Kusatsu, Japan). For functional validation of the CRE and HBE sites identified in the proximal region of IGF-I promoter, sequence truncation, or loss-of-function mutation of these cis-acting elements was introduced in the IGF-I promoter within pIGF1.Luc construct using PCR-based deletion or Quick Change Site-Directed Mutagenesis Kit (Agilent, Santa Clara, CA). For promoter activity expression, αT3 cells seeded at ~0.1 × 10^6^ cells/ml/well in 24-well plate were transfected in Opti-MEM for 6 h with lipofectamine (Invitrogen) in the presence of 200 ng pIGF1.Luc construct (with or without 5′ deletion of IGF-I promoter or CRE and HBE truncation/mutation), 50 ng glucagon receptor expression vector, 10 ng TK-Renilla (internal control), and 20 ng pEGFP-N1 (for tracking transfection efficiency) with pcDNA/Zeo as the carrier DNA to make up a total of 400 ng DNA per transfection. After a 15-h incubation for recovery, the cells were treated with glucagon for the dose and duration as indicated. To study the functional role of CREB and HNF1α in glucagon-induced IGF-I promoter activation, parallel transfection with the expression vector for carp CREB or HNF1α was also conducted prior to glucagon exposure. After drug treatment, αT3 cells were dissolved in passive lysis buffer (Promega) and the cell lysate prepared was subjected to luciferase activity measurement using a Dual-Glo^®^ Luciferase Assay Kit (Promega, Madison, WI) as described previously (34).

### Data Transformation and Statistical Analysis

For real-time PCR of IGF-I, CREB, and HNF1α mRNA as well as IGF-I promoter and primary transcript, standard curves constructed with plasmids carrying the respective gene targets with dynamic range of ≥10^5^, amplification efficiency ≥98%, and correlation co-efficiency ≥0.95 were used for data calibration with the RotorGene Q.Rex software (Qiagen). Since the expression of 18S RNA (as internal control) did not show significant changes in our experiments, the raw data for respective gene targets were simply transformed as a percentage of the mean value in the control group (as “%Ctrl”). For the transfection studies using Luc reporters, luciferase activities expressed in αT3 cells were measured in terms of “arbitrary light unit.” To control for the variations in transfection efficiency between wells, the firefly luciferase activity conferred by the target gene promoter was normalized as a ratio of the Renilla luciferase activity caused by co-transfection of TK-Renilla (as internal control) in the same sample (as “LUC activity ratio”). Similar to the real-time PCR data, the normalized data for luciferase activity were then expressed as a percentage of the mean value in the control prior to statistical analysis. Data presented (mean ± SEM) were pooled results from 4 to 6 separate experiments and analyzed with one-way ANOVA (for dose-dependence/interaction studies with pharmacological inhibitors) or two-way ANOVA (for time course) followed by Newman-Keuls *post-hoc* test. Differences were considered as significant at *p* < 0.05.

## Results

### Structural Characterization, Tissue Distribution, and Functional Validation of Carp HNF1α

**Figure 1:**
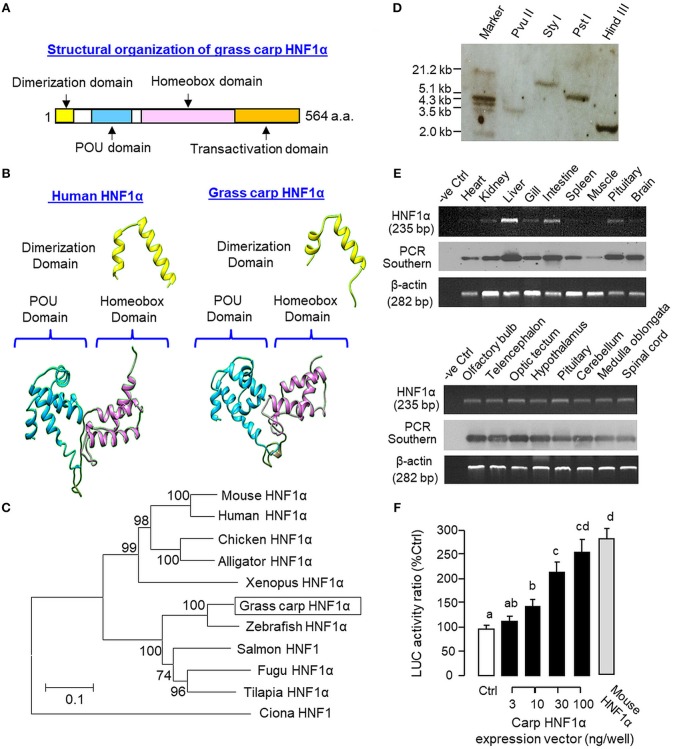
Structural characterization, gene copy number, tissue distribution, and functional validation of grass carp HNF1α. **(A)** Structural organization of carp HNF1α a.a. sequence into four signature motifs, including the dimerization domain, POU domain, homeobox domain, and transactivation domain. **(B)** 3D Protein modeling of the dimerization domain, POU domain, and homeobox domain of carp HNF1α using the corresponding structures of their human counterparts as the template with SWISS-MODEL program. **(C)** Phylogenetic analysis of carp HNF1α cDNA sequence using the neighbor-joining method with MEGA 6.0 with the corresponding sequences identified in other species. The scale bar represents the evolutionary distance and the values for individual nodes of the guide tree are the percentage based on 1,000 bootstraps. **(D)** Copy number of HNF1α gene in the carp genome. Southern blot was conducted in genomic DNA after digestion with Pvu II, Sty I, Pst I, and Hind III, respectively, using a DIG-labeled cDNA probe for HNF1α. **(E)** Tissue expression profiling of HNF1α with RT-PCR. Total RNA was isolated from selected tissues and brain areas and subjected to RT-PCR using primers specific for carp HNF1α. The authenticity of PCR products was confirmed by PCR Southern and RT-PCR for β actin was used as the internal control. **(F)** Functional expression of carp HNF1α in αT3 cells. The ORF of grass carp HNF1α was subcloned into the eukaryotic expression vector pcDNA3.1 and transfected into αT3 cells with the Luc reporter pGL_3_.HNF1α.Luc with tandem repeats of HBE sites in its 5′promoter. In this study, parallel transfection with the expression vector for mouse HNF1α was used as a positive control. Data presented are expressed as mean ± SEM and groups denoted by different letters represent a significant difference at *p* < 0.05 (ANOVA followed by Newman-Keuls test).

As a first step to study the functional role of HNF1α in IGF-I gene expression in grass carp, the structural identity of grass carp HNF1α was established by 3′/5′ RACE using total RNA from the liver as the template (Supplemental Figure 1). As revealed by the cDNA sequence obtained, the ORF of carp HNF1α is 1,695 bp in size encoding a 564 a.a. protein (with MW of ~63 kDa). The deduced protein is composed of an N-terminal dimerization domain followed by the POU and homeobox domains in the central region and a transactivation domain in the C-terminal tail (Figure 1A). Sequence alignment at the protein level also confirms that the carp HNF1α sequence is highly homologous to those reported in other vertebrates, especially in the POU domain (76.2–95.2%) and homeobox domain (73.2–97.9%; Supplemental Figure 2). Using 3D protein modeling, the spatial arrangement of the α helical structures within the dimerization domain, POU domain, and homeobox domain of the carp HNF1α was found to be highly comparable, if not identical, with their counterparts in human HNF1α (Figure 1B). In parallel phylogenetic analysis using neighbor-joining method, the nucleotide sequence of carp HNF1α could also be clustered within the clade of fish HNF1α with a very close evolution relationship with zebrafish HNF1α (Figure 1C).

To deduce the copy number of HNF1α gene in grass carp, Southern blot was performed in genomic DNA with digestion of Pvu II, Sty I, Pst I, and Hind III, respectively. As shown in Figure 1D, a single band was revealed in different samples after hybridization with the DIG-labeled probe for HNF1α, implying that HNF1α exists as a single-copy gene in the carp genome. For tissue expression of HNF1α, RT-PCR was also performed in selected tissues and brain areas. In this case, PCR signals for HNF1α were found to be ubiquitously expressed in tissues including the heart, kidney, liver, gill, intestine, spleen, muscle, and pituitary (Figure 1E, upper panels) as well as in brain areas including the olfactory bulb, telencephalon, optic tectum, hypothalamus, cerebellum, medulla oblongata, and spinal cord (Figure 1E, lower panels). The highest level of HNF1α signal was located in the liver while a lower extent of HNF1α expression could also be noted in the intestine, pituitary, kidney, gill, and different brain areas. Using LC/MS/MS, peptide fragments originated from HNF1α were consistently detected in protein samples prepared from the carp liver with trypsin digestion (protein coverage by peptides identified: 84.2% for the peptides with ≥75% confidence and 57.3% for the peptides with 99% confidence; Supplemental Figure 3). To test if the newly cloned cDNA indeed encodes a HNF1α protein with bioactivity, functional expression of carp HNF1α was conducted in αT3 cells transfected with a Luc reporter carrying tandem repeats of HBE sites in its 5′ promoter. Similar to the study with mouse HNF1α expression (as a positive control), transfection with the expression vector for carp HNF1α was effective in inducing luciferase activity expression in a dose-dependent manner (Figure 1F).

### Effects of Glucagon on HNF1α, CREB, and IGF-I mRNA Expression in Carp Hepatocytes

**Figure 2:**
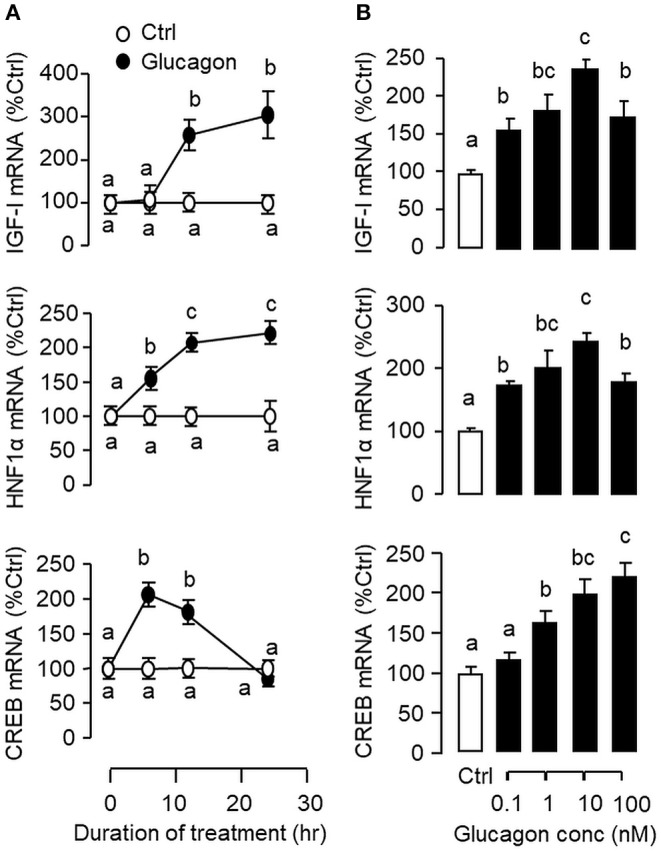
Glucagon-induced IGF-I, HNF1α, and CREB gene expression in primary culture of grass carp hepatocytes. **(A)** Time course and **(B)** Dose dependence of glucagon stimulation on IGF-I, HNF1α, and CREB mRNA expression in carp hepatocytes. For the time course experiment, the dose of glucagon used was fixed at 10 nM for the duration as indicated, while the duration of drug treatment was fixed at 12 h for the dose-response study. After glucagon induction, total RNA was isolated from individual wells and used for real-time PCR using primers for carp IGF-I, HNF1α, and CREB, respectively. Data presented are expressed as mean ± SEM and groups denoted by different letters represent a significant difference at *p* < 0.05 (ANOVA followed by Newman-Keuls test).

**Figure 3:**
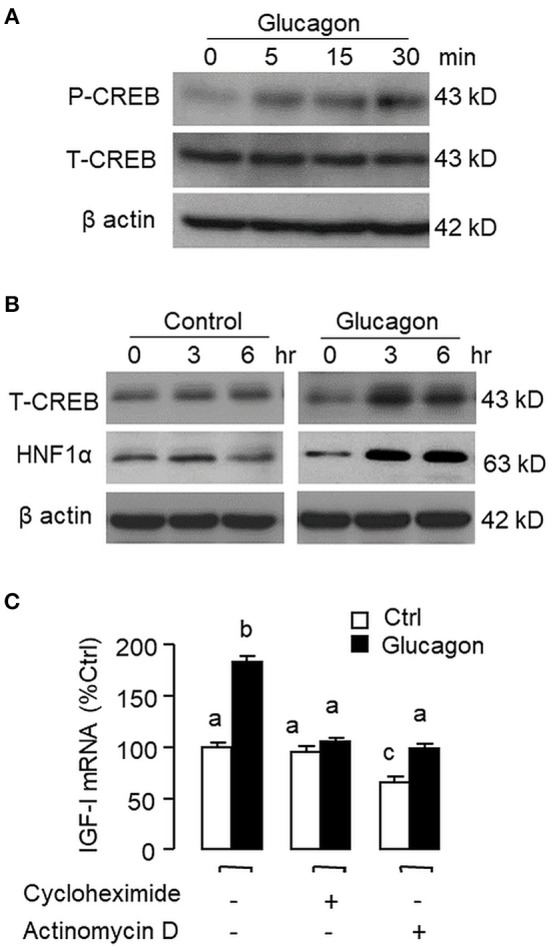
Glucagon-induced HNF1α and CREB expression in carp hepatocytes and function role of gene transcription and protein synthesis in the corresponding responses for IGF-I transcript. **(A)** Short-term treatment of glucagon (10 nM, up to 30 min) on CREB phosphorylation. **(B)** Long-term treatment of glucagon (10 nM, up to 6 h) on HNF1α and CREB protein expression. In these experiments, cell lysate was prepared from carp hepatocytes after drug treatment at the time points as indicated and subjected to Western blot using antibodies for phosphorylated CREB (as “P-CREB”) and total protein for CREB (as “T-CREB”) and HNF1α, respectively. Parallel blotting for β actin was also conducted to serve as the internal control. **(C)** Blocking gene transcription and protein synthesis on glucagon-induced IGF-I mRNA expression. Hepatocytes were challenged with glucagon (10 nM) for 12 h in the presence of the transcriptional inhibitor actinomycin D (8 μM) or protein synthesis inhibitor cycloheximide (10 μg/ml). After drug treatment, total RNA was isolated from individual wells and used for real-time PCR with primers specific for IGF-I transcript.

To establish the temporal relationship of HNF1α and CREB expression related to IGF-I regulation by glucagon in the carp liver, time-course study was conducted in primary culture of carp hepatocytes with glucagon treatment. As shown in Figure 2A, glucagon could elevate IGF-I mRNA levels with parallel rises of HNF1α and CREB transcripts. Of note, the significant elevations in HNF1α and CREB signals (occurred at 6 h) were detected well before the notable rise in IGF-I gene expression (occurred at 12 h). By fixing the duration of drug treatment at 12 h, increasing levels of glucagon were also found to induce IGF-I, HNF1α, and CREB mRNA expression in a dose-dependent manner (Figure 2B). In parallel experiments, short-term stimulation with glucagon (up to 30 min) could trigger rapid phosphorylation of CREB in carp hepatocytes (Figure 3A) while a longer duration of glucagon treatment (up to 6 h) was effective in increasing total protein of CREB and HNF1α, respectively (Figure 3B). In the same cell model, glucagon-induced IGF-I mRNA expression was also sensitive to the blockade of gene transcription by actinomycin D or inhibiting protein synthesis using cycloheximide (Figure 3C), which raises the possibility that HNF1α and CREB transcript expression/protein production may play a role in IGF-I regulation by glucagon.

### Signal Transduction for IGF-I, HNF1α, and CREB mRNA Expression Induced by Glucagon

**Figure 4:**
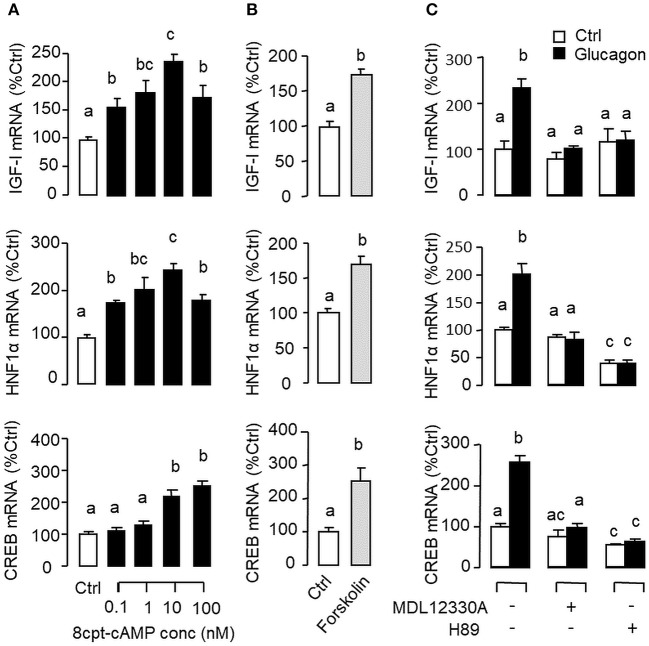
Functional role of the AC/cAMP/PKA pathway in glucagon-induced IGF-I, HNF1α, and CREB transcript expression at the hepatic level. Effects of **(A)** increasing functional levels of cAMP using the membrane-permeant cAMP analog 8cpt-cAMP (0.1–100 nM) or **(B)** stimulating cAMP synthesis with the adenylate cyclase (AC) activator forskolin (1 μM) on IGF-I, HNF1α, and CREB mRNA expression in carp hepatocytes. In parallel study, glucagon induction (10 nM) was also tested with co-treatment of the AC inhibitor MDL12330A (20 μM) or PKA inactivator H89 (20 μM) **(C)**. In these experiments, the duration of drug treatment was fixed at 12 h. After that, total RNA was isolated from individual wells and used for real-time PCR with primers for IGF-I, HNF1α, and CREB, respectively.

**Figure 5:**
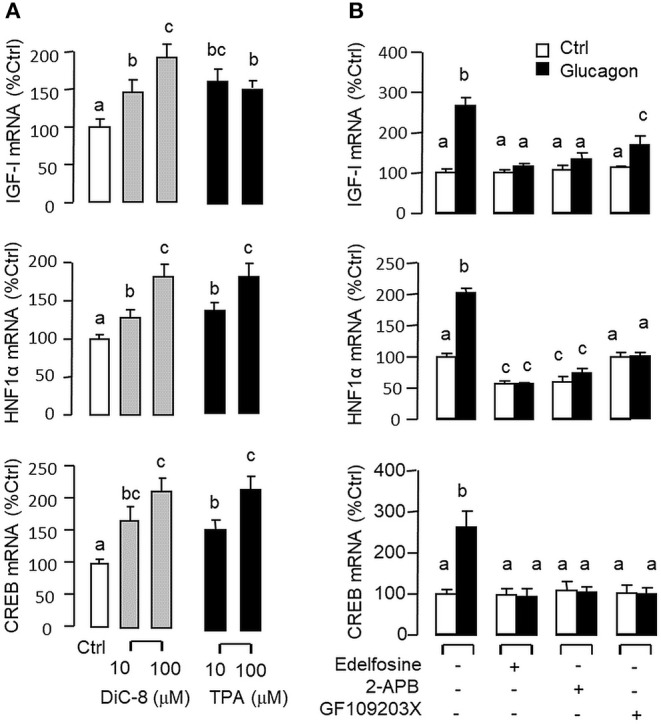
Functional role of the PLC/IP_3_/PKC pathway in glucagon-induced IGF-I, HNF1α, and CREB transcript expression at the hepatic level. Effects of increasing functional levels of DAG using the DAG analog DiC-8 (10–100 nM) or stimulating PKC activity using the PKC activator TPA (10–100 M) on IGF-I, HNF1α, and CREB mRNA expression in carp hepatocytes **(A)**. In parallel experiments, glucagon treatment (10 nM) was also tested in the presence of the PLC inhibitor Edelfosine (20 μM), IP_3_ receptor blocker 2-APB (100 μM) or PKC inactivator GF109203X (10 μM) **(B)**. In these studies, the duration of drug treatment was fixed at 12 h. After that, total RNA was isolated from individual wells and used for real-time PCR with primers for IGF-I, HNF1α, and CREB, respectively.

**Figure 6:**
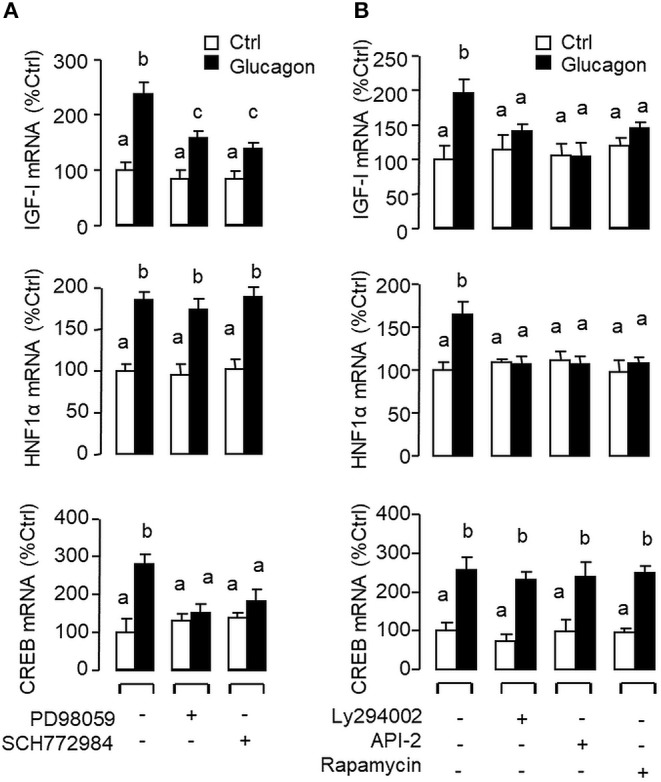
Functional role of the MAPK and PI3K/Akt cascades in glucagon-induced IGF-I, HNF1α, and CREB transcript expression at the hepatic level. Carp hepatocytes were treated with glucagon (10 nM) for 12 h in the presence of **(A)** the MEK_1/2_ inhibitor PD98059 (10 μM) or ERK_1/2_ inhibitor SCH772984 (10 nM) or **(B)** the PI3K inhibitor Ly294002 (10 μM), Akt blocker API-2 (2 μM), or mTOR inactivator Rapamycin (20 nM). After drug treatment, total RNA was isolated from individual wells and subjected to real-time PCR with primers for IGF-I, HNF1α, and CREB, respectively.

Given that glucagon receptor is known to be functionally coupled with cAMP/PKA and PLC/IP_3_/PKC pathways (35, 36), the potential involvement of the two post-receptor signaling cascades in IGF-I regulation by glucagon in the carp liver was examined. In carp hepatocytes, the stimulatory effects of glucagon on IGF-I, HNF1α, and CREB mRNA expression were mimicked by treatment with the cAMP analog 8cpt-cAMP (Figure 4A) or by activating cAMP synthesis using the adenylate cyclase (AC) activator forskolin (Figure 4B). Transcript expression of IGF-I, HNF1α, and CREB induced by glucagon, in contrast, could be blocked by co-treatment of the AC inhibitor MDL12330A or PKA inhibitor H89 (Figure 4C). In parallel studies, IGF-I, HNF1α, and CREB gene expression could also be up-regulated by treatment with the DAG analog DiC-8 or PKC activator TPA (Figure 5A) whereas the corresponding responses induced by glucagon were reduced/negated by the PLC inhibitor edelfosine, IP_3_ receptor blocker 2-APB or PKC inactivator GF109203X (Figure 5B). In different cell models, glucagon actions mediated by MAPK (37, 38) and/or PI3K/Akt cascades (39) have also been documented. In our studies with carp hepatocytes, glucagon-induced IGF-I, and CREB but not HNF1α transcript expression could be reduced/abolished by treatment with the MEK_1/2_ inhibitor PD98059 or ERK_1/2_ inhibitor SCH772984 (Figure 6A). However, parallel blockade with the PI3K inhibitor Ly294002, Akt blocker API-2, or mTOR inactivator rapamycin was effective in abating the actions of glucagon on IGF-I and HNF1α mRNA levels but with no effects on CREB gene expression (Figure 6B).

### Signaling Crosstalk in Glucagon-Induced IGF-I, HNF1α, and CREB mRNA Expression

**Figure 7:**
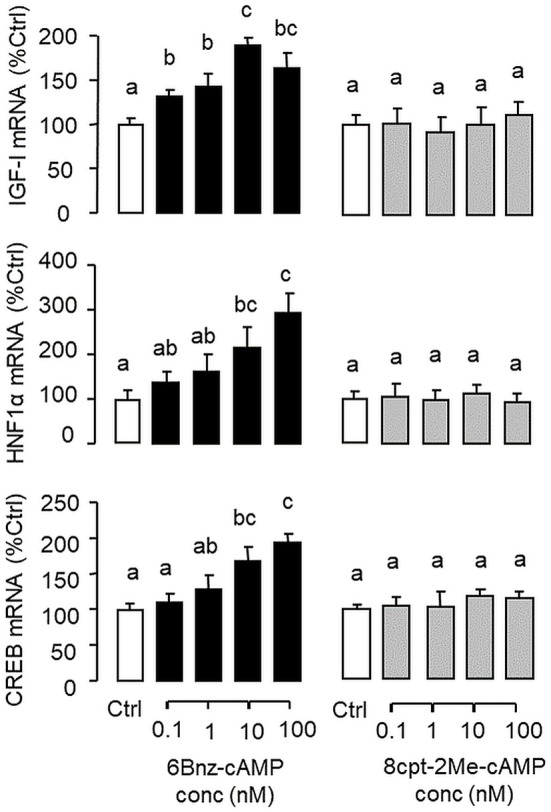
Functional role of PKA and Epac activation on IGF-I, HNF1α, and CREB transcript expression at the hepatic level. Carp hepatocytes were treated for 12 h with increasing concentrations (0.1–100 nM) of the PKA-specific cAMP analog 6Bnz-cAMP or Epac-specific cAMP analog 8cpt-2Me-cAMP. After drug treatment, total RNA was isolated from individual wells and subjected to real-time PCR with primers for IGF-I, HNF1α, and CREB, respectively.

**Figure 8:**
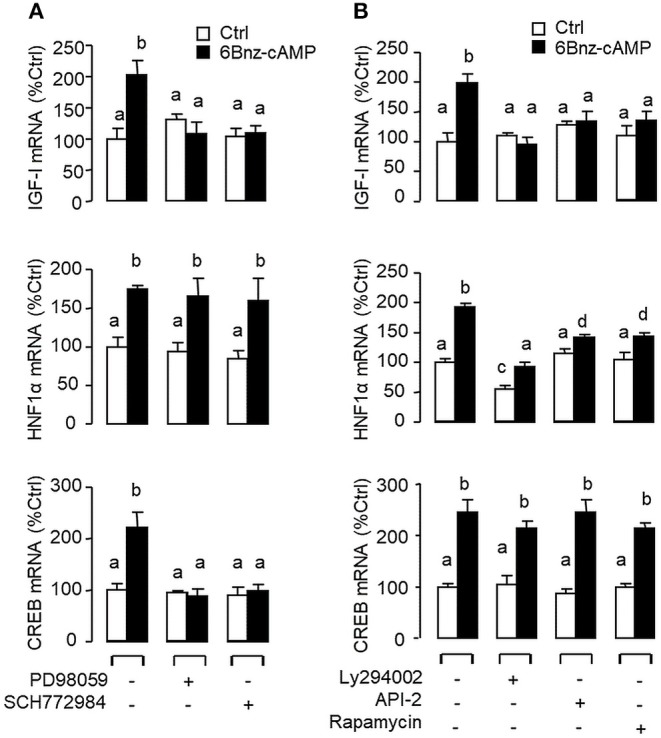
Functional coupling of MAPK and PI3K/Akt cascades with PKA activation in IGF-I, HNF1α, and CREB mRNA expression at the hepatic level. Carp hepatocytes were exposed to the PKA-specific cAMP analog 6Bnz-cAMP (100 nM) for 12 h with co-treatment of **(A)** the MEK_1/2_ inhibitor PD98059 (10 μM) or ERK_1/2_ inhibitor SCH772984 (10 nM), or **(B)** the PI3K inhibitor Ly294002 (10 μM), Akt blocker API-2 (2 μM), or mTOR inactivator Rapamycin (20 nM). After drug treatment, total RNA was isolated from individual wells and used for real-time PCR with primers for IGF-I, HNF1α, and CREB, respectively.

**Figure 9:**
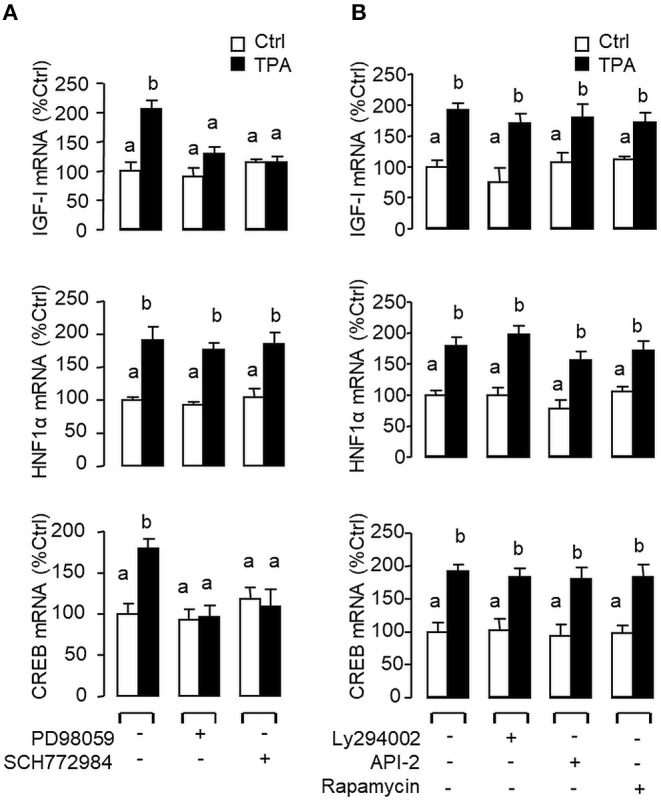
Functional coupling of MAPK and PI3K/Akt cascades with PKC activation in IGF-I, HNF1α, and CREB mRNA expression at the hepatic level. Carp hepatocytes were exposed to the PKC activator TPA (10 μM) for 12 h in the presence of **(A)** the MEK_1/2_ inhibitor PD98059 (10 μM) or ERK_1/2_ inhibitor SCH772984 (10 nM), or **(B)** the PI3K inhibitor Ly294002 (10 μM), Akt blocker API-2 (2 μM), or mTOR inactivator Rapamycin (20 nM). After that, total RNA was isolated from individual wells and subjected to real-time PCR with primers for IGF-I, HNF1α, and CREB, respectively.

In mammals, functional crosstalk of cAMP with MAPK cascades through Epac activation of Rap1 is well-documented (40), e.g., for glucose homeostasis via insulin/glucagon signaling (41). To evaluate the functional role of Epac vs. PKA in cAMP dependence of glucagon actions, carp hepatocytes were exposed to increasing levels of the cAMP analogs 6Bnz-cAMP and 8cpt-2Me-cAMP, which are known to be specific for PKA and Epac activation, respectively (Figure 7). In this case, 6Bnz-cAMP but not 8cpt-2Me-cAMP was found to mimic the dose dependence of glucagon on IGF-I, HNF1α, and CREB mRNA expression. To shed light on the possible interaction of cAMP/PKA and PLC/IP_3_/PKC pathways with MAPK and PI3K/Akt cascades in mediating glucagon actions at the hepatic level, carp hepatocytes were challenged with the PKA-specific cAMP analog 6Bnz-cAMP (Figure 8) or PKC activator TPA (Figure 9) in the presence of the pharmacological inhibitors targeting MAPK and PI3K/Akt cascades. Similar to the preceding studies with glucagon, 6Bnz-cAMP consistently up-regulated IGF-I, HNF1α, and CREB mRNA levels and the stimulatory effects on IGF-I and CREB but not HNF1α were totally blocked by co-treatment with the MEK_1/2_ inhibitor PD98059 or ERK_1/2_ inhibitor SCH772984 (Figure 8A). In parallel experiments, 8Bnz-cAMP induction of IGF-I and HNF1α mRNA expression were also reduced/negated by the PI3K inhibitor Ly294002, Akt blocker API-2, or mTOR inactivator rapamycin. Similar treatment, however, did not affect the CREB signals induced by 8Bnz-cAMP (Figure 8B). For PKC crosstalk with MAPK and/or PI3K/Akt cascades, TPA-induced IGF-I and CREB, but not HNF1α mRNA expression could be abated by inhibiting MEK_1/2_ with PD98059 or blocking ERK_1/2_ with SCH772984 (Figure 9A). Interestingly, the stimulatory effects on IGF-I, HNF1α, and CREB expression induced by TPA were not altered by the PI3K inhibitor LY294002, Akt blocker API-2 or mTOR inactivator rapamycin (Figure 9B).

### Glucagon-Induced Pol-II Recruitment and IGF-I Primary Transcript Expression

**Figure 10:**
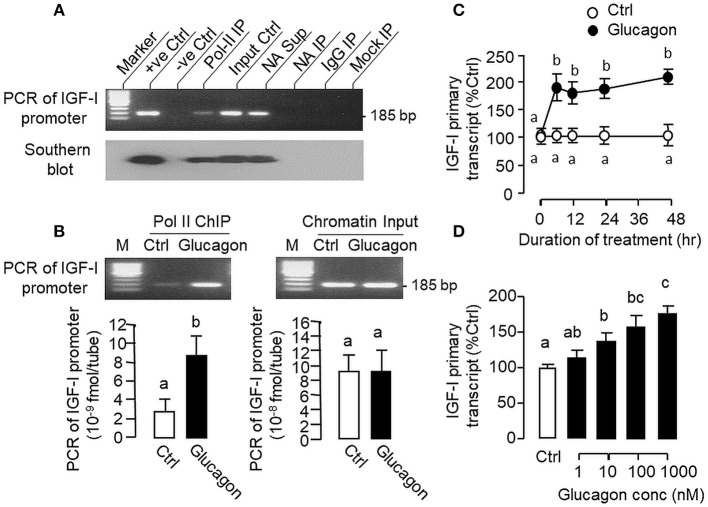
Glucagon-induced Pol-II recruitment to IGF-I promoter and production of IGF-I primary transcript in carp hepatocytes. **(A)** Pol-II binding with IGF-I promoter revealed by PCR coupled with chromatin immunoprecipitation (ChIP). Chromatin samples with fragment size of 0.5–0.8 kb after sonication were prepared from carp hepatocytes and used for ChIP with the antibody for RNA Po-II (as “Pol-II IP”). After decrosslinking, PCR was performed using primers covering the proximal region of the carp IGF-I promoter. In this experiment, genomic DNA (as “+ve Ctrl”), chromatin input (as “Input Ctrl”) and “supernatant” of chromatin sample after IP with no antibody (as “NA Sup”) were used as the positive control. PCR without adding the template (as “–ve Ctrl”) or with chromatin samples after IP with “No Antibody” (as “NA IP”)/parallel ChIP with antibody for mouse IgG (as “IgG IP”) or without chromatin input (as “Mock IP”) were used as the negative control. The authenticity of PCR products detected was also confirmed with Southern blot using a DIG-labeled probe covering the same region of the IGF-I promoter. **(B)** Glucagon-induced Pol-II recruitment to IGF-I promoter in carp hepatocytes. Hepatocytes were challenged for 1 h with glucagon (10 nM) and subjected to chromatin preparation followed by ChIP with Pol-II antibody. After that, real-time PCR with primers covering the proximal region of IGF-I promoter was performed for quantitation of IGF-I promoter pulled down with RNA Pol-II. Real-time PCR for IGF-I promoter was also conducted in the chromatin input prior to Pol-II ChIP to serve as the loading control. **(C)** Time course and **(D)** Dose dependence of glucagon treatment on IGF-I primary transcript expression in carp hepatocytes. For the time course experiment, the dose of glucagon used was fixed at 100 nM for the duration as indicated, while the duration of drug treatment was fixed at 12 h for the dose-response study. After that, total RNA was isolated, digested with DNase I to remove genomic DNA contamination, and subjected to real-time PCR using primers covering the junction of exon III and intron III of carp IGF-I gene.

During the process of gene transcription, recruitment of RNA Pol-II to target gene promoter is a prerequisite for primary transcript production (42) and subsequent intron splicing can lead to formation of mature mRNA (43). To investigate the role of gene transcription in glucagon-induced IGF-I mRNA expression at the hepatic level, Pol-II ChIP coupled to PCR detection of IGF-I promoter was performed in carp hepatocytes (Figure 10A). In chromatin sample pulled down by immunoprecipitation (IP) with the Pol-II antibody (as “Pol-II IP”), a single PCR product of 185 bp (covering −116 to +69 of IGF-I gene) was detected with primers flanking the proximal region of carp IGF-I promoter and the authenticity of the PCR product was confirmed with Southern blot using a DIG-labeled probe covering the same region. Although the 185 bp PCR product was also noted in parallel PCR with genomic DNA (as “+ve Ctrl”), chromatin input (as “Input Ctrl”) and “supernatant” of chromatin with “No Antibody (NA)” IP (as “NA Sup”), PCR signals were not observed in chromatin samples after IP with no antibody (as “NA IP”) or with mouse IgG (as “IgG IP”) as well as in mock IP without chromatin input (as “Mock IP”), indicating that the ChIP-PCR signal for IGF-I promoter is highly specific in our system. In parallel study with Pol-II ChIP coupled to real-time PCR for IGF-I promoter, prior treatment of hepatocytes with glucagon was found to enhance the PCR signal for IGF-I promoter pulled down with the Pol-II antibody (Figure 10B), implying that glucagon can promote Pol-II recruitment to IGF-I promoter at hepatic level. Consistent with the IGF-I mRNA responses observed in preceding studies, glucagon treatment was also effective in up-regulating the levels of IGF-I primary transcript in carp hepatocytes in a time- (Figure 10C) and dose-dependent manner (Figure 10D).

### Glucagon-Induced IGF-I Promoter Activity and Functional Role of HNF1α and CREB

**Figure 11:**
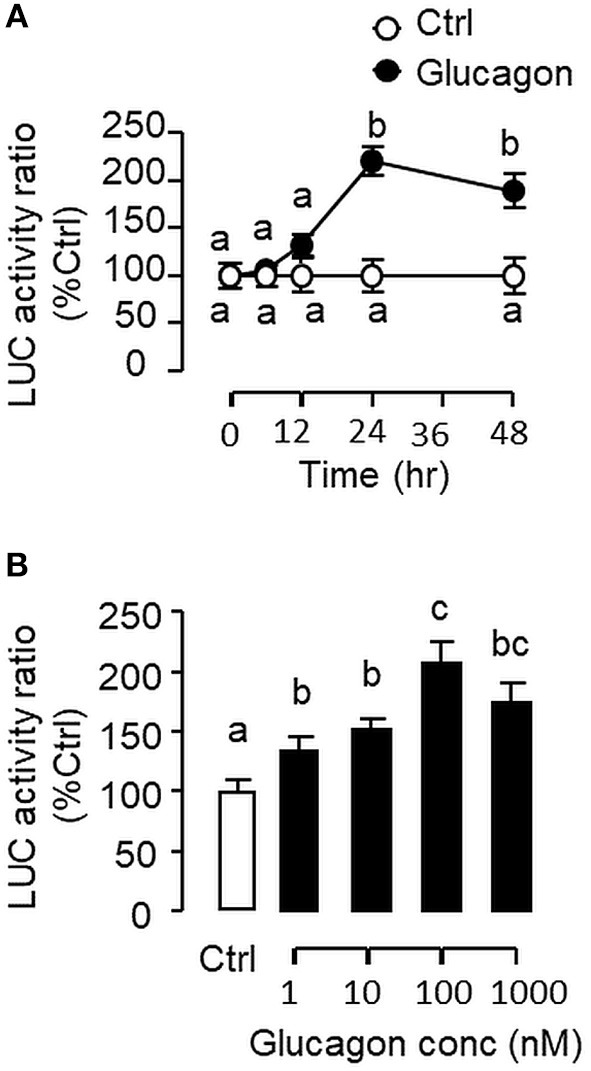
Glucagon-induced IGF-I promoter activity expressed in αT3 cells. **(A)** Time course and **(B)** Dose dependence for glucagon induction of IGF-I promoter activity of carp origin. Transfection studies was performed in αT3 cells with pIGF1(-1077).Luc carrying a 1,077 bp 5′ promoter of the carp IGF-I gene. Glucagon treatment was initiated after 15 h incubation to allow for recovery after transfection. For time course experiment, the dose of glucagon used was fixed at 100 nM for the duration as indicated, while the duration of treatment was fixed at 24 h for the dose-response study. After glucagon treatment, cell lysate was prepared from αT3 cells and used for luciferase activity measurement.

**Figure 12:**
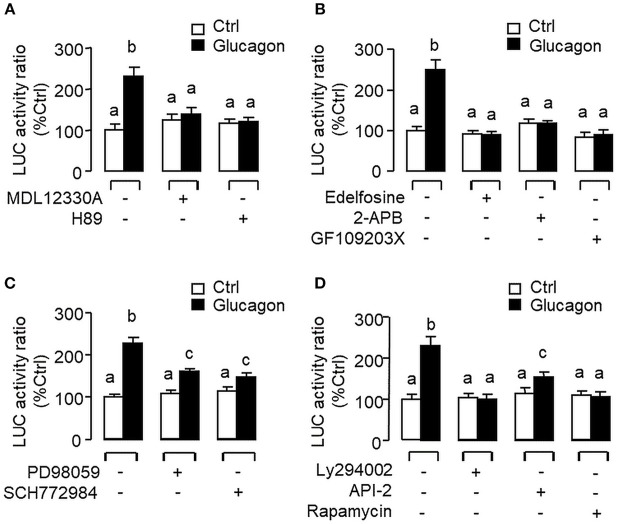
Post-receptor signaling for glucagon-induced IGF-I promoter activity expressed in αT3 cells. After transfection with pIGF1(-1077).Luc, αT3 cells were treated with glucagon (100 nM) for 24 h in the presence of **(A)** the AC inhibitor MDL12330A (20 μM) or PKA blocker H89 (20 μM), **(B)** the PLC inhibitor Edelfosine (20 μM), IP_3_ receptor blocker 2-APB (100 μM) or PKC inactivator GF109203X (10 μM), **(C)** the MEK_1/2_ inhibitor PD98059 (10 μM) or ERK_1/2_ inhibitor SCH772984 (10 nM), or **(D)** the PI3K inhibitor Ly294002 (10 μM), Akt blocker API-2 (2 μM), or mTOR inactivator Rapamycin (20 nM). After drug treatment, cell lysate was prepared and used for luciferase activity measurement.

**Figure 13:**
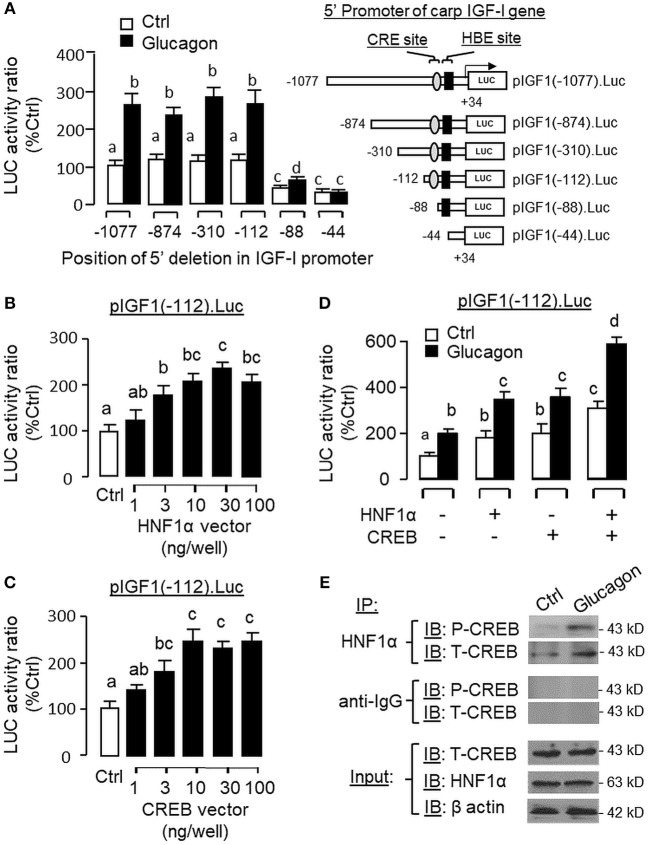
Function role of HNF1α and CREB in glucagon-induced IGF-I promoter activity in αT3 cells. **(A)** Mapping of glucagon responsive sequence to the proximal promoter of IGF-I gene with CRE and HBE sites. 5′ Deletion was conducted in the 1,077 bp promoter of pIGF1(-1077).Luc to generate a series of 5′ deleted Luc reporters with decreasing lengths of IGF-I promoter from position −874 to −44. These 5′ deletion constructs were then used for transfection study in αT3 cells followed by 24 h treatment with glucagon (100 nM). Over-expression of **(B)** HNF1α and **(C)** CREB on IGF-I promoter activation. Co-transfection was performed in αT3 cells with pIGF1(-112).Luc (with CRE and HBE sites in proximal promoter) together with increasing levels of the expression vector for carp HNF1α or CREB as indicated. **(D)** Functional interaction of HNF1α and CREB on glucagon-induced IGF-I promoter activity expressed in αT3 cells. In parallel study with pIGF1(-112).Luc, similar transfection was conducted with the HNF1α vector alone (30 ng/ml), CREB vector alone (30 ng/ml) or co-transfection with the two vectors (30 ng/well each). After that, the cells were treated with glucagon (100 nM) for 24 h. In our experiments with IGF-I promoter, cell lysate was prepared from αT3 cells after the drug treatment/transfection with expression vectors and used for luciferase activity measurement. **(E)** Protein:protein interaction of HNF1α and CREB induced by glucagon in carp hepatocytes. Immunoprecipitation (IP) was performed in cell lysate prepared from carp hepatocytes after 1-h treatment with glucagon (10 nM) using the antibody for HNF1α. After that, the protein pull-down was subjected to immunoblotting (IB) with antibodies for phosphorylated and total protein of CREB (as “P-CREB” and “T-CREB,” respectively). In this experiment, parallel IP with the antibody for mouse IgG (as “anti-IgG”) was used as the negative control while the corresponding IB for HNF1α, T-CREBk, and β actin were conducted in the cell lysate prior to the pull-down with HNF1α antibody to serve as the input control.

To evaluate the role of promoter activation in glucagon-induced IGF-I gene transcription, αT3 cells with glucagon receptor expression were used as the host cells to examine glucagon activation of IGF-I promoter of carp origin. After transfection with pIGF1(-1077).Luc carrying a ~1 kb 5′ promoter of carp IGF-I gene, luciferase activity expressed in αT3 cells could be elevated in a time- (Figure 11A) and dose-related fashion with glucagon treatment (Figure 11B). In parallel studies, the luciferase activity induced by glucagon could be reduced/negated by (i) inhibiting the cAMP/PKA pathway using the AC blocker MDL12330A or PKA inhibitor H89 (Figure 12A), (ii) blocking the PLC/IP_3_/PKA pathway with the PLC inhibitor edelfosine, IP_3_ receptor blocker 2-APB or PKC inactivator GF109203X (Figure 12B), (iii) impeding MAPK signaling using the MEK_1/2_ inhibitor PD98059 or ERK_1/2_ inhibitor SCH772984 (Figure 12C), and (iv) inactivating the PI3K/Akt cascade with the PI3K inhibitor LY294002, Akt blocker API-2 or mTOR inactivator rapamycin (Figure 12D). These results imply that the αT3 cells can faithfully mimic the post-receptor signaling for IGF-I regulation by glucagon in carp hepatocytes. To map the responsive sequence in the IGF-I promoter for glucagon action, 5′ deletion analysis was conducted in the promoter of pIGF1(-1077).Luc to generate a series of deletion mutants with decreasing lengths of IGF-I promoter from −874 to −44 (Figure 13A). Transfection with these mutants revealed that both the basal and glucagon-induced luciferase activity were not affected by 5'deletion up to position −112. A further deletion from position −112 to −88 with a CRE site (TGACGTTA), however, was effective in attenuating both basal and glucagon-induced luciferase activity. Of note, the stimulatory effect of glucagon was totally ablated by further removal of the region from −88 to −44 containing a HBE site (CTTAATGAGTAAC). These results indicate that the proximal region downstream of position −112 with the CRE and HBE sites is essential for IGF-I promoter activation caused by glucagon.

**Figure 14:**
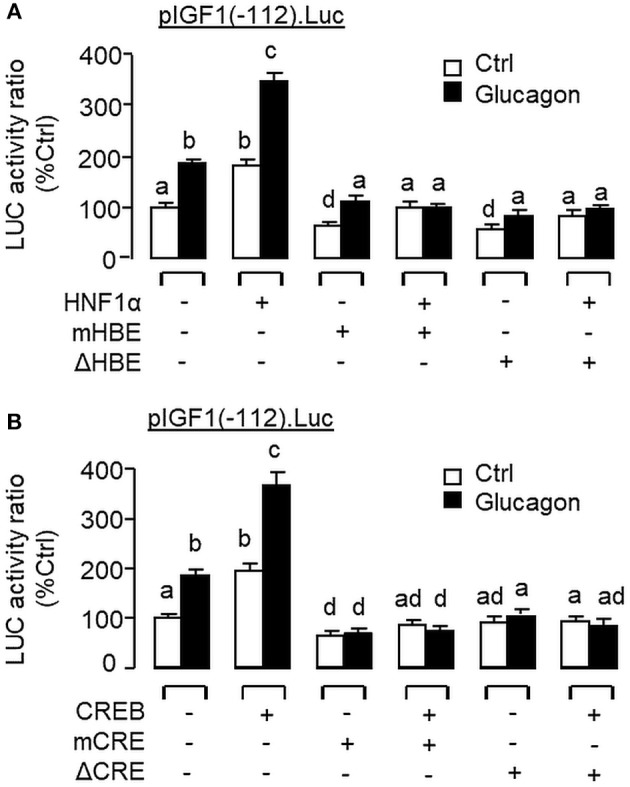
Functional role of HBE and CRE sites within the proximal promoter of IGF-I gene in glucagon-induced IGF-I promoter activity in αT3 cells. Site-directed mutation and PCR-based truncation were conducted in **(A)** the HBE site (as “mHBE” and “ΔHBE,” respectively) and **(B)** CRE site (as “mCRE” and “ΔCRE,” respectively) within the IGF-I promoter of pIGF1(-112).Luc. After that, the constructs with HBE and CRE mutation/truncation were used for co-transfection studies in αT3 cells together with the expression vector for HNF1α and CREB, respectively, followed by a 24-h treatment with glucagon (100 nM). In these experiments, parallel transfection with the wild type pIGF1(-112).Luc was used as the control. After drug treatment, cell lysate was prepared and used for luciferase activity measurement.

To examine the functional role of HNF1α and CREB in IGF-I promoter activation, over-expression of HNF1α and CREB were performed in αT3 cells transfected with pIGF1(-112).Luc carrying a 112 bp IGF-I promoter with the CRE and HBE sites in the proximal region. In this case, transfection with the HNF1α expression vector (Figure 13B) or CREB expression vector (Figure 13C) was effective in stimulating luciferase activity expression in a dose-dependent manner. Over-expression of HNF1α or CREB not only could increase basal but also enhance glucagon-induced luciferase activity and these stimulatory effects were found to be additive with co-expression of the two transcription factors (Figure 13D). In carp hepatocytes, Western blot for CREB in the protein pull-down (IP) using HNF1α antibody revealed that the immunoblotting (IB) signals for phosphorylated and total protein of CREB could be enhanced by glucagon treatment (Figure 13E). Since no CREB signals could be noted in the protein pull-down by IgG antibody, our results suggest that the association of HNF1α with activated CREB is specific and can be induced by glucagon at the hepatic level. To shed light on the functionality of the HBE and CRE sites located in the proximal region of IGF-I promoter, site-directed mutagenesis and PCR-based truncation of these cis-acting elements were also conducted in pIGF1(-112).Luc. Similar to our preceding study, both basal and glucagon-induced luciferase activity were up-regulated in αT3 cells co-transfected with pIGF1(-112). Luc and HNF1α expression vector and these stimulatory actions were reduced/obliterated with mutation (mHBE, CATATGGAGTAAC) or truncation of the HBE site (ΔHBE) within the IGF-I promoter (Figure 14A). In parallel study, similar enhancement in basal and glucagon-induced luciferase activity were also noted with co-transfection of pIGF1(-112).Luc and CREB expression vector. Again, these stimulatory effects could be blocked by mutation (mCRE, TACGGTTA) or truncation of the CRE site (ΔCRE) within the proximal region of IGF-I promoter (Figure 14B).

## Discussion

Among the transcription factors involved in IGF-I gene expression, the role of HNF1α appears to be well-conserved and its binding site(s), namely HBE, can be identified within the IGF-I promoter from fish to mammals (22). In previous studies, over-expression of HNF1α was shown to elevate basal and/or confer GH inducibility for IGF-I gene transcription while mutation of HBE site(s) can suppress GH-induced IGF-I promoter activation, e.g., in human (44) and fish models including the salmon (23) and common carp (25). However, endocrine regulation of HNF1α expression as a means to modify/control IGF-I gene transcription has not been examined and remains an unexplored area for IGF-I research. In our study with grass carp, a CRE site was located right next to the HBE site in the proximal promoter of IGF-I gene and the close proximity of the two cis-acting elements raises the possibility that HNF1α may act with CREB to regulate IGF-I gene transcription. As a first step to investigate HNF1α regulation related to glucagon modulation of IGF-I expression in carp model, grass carp HNF1α was cloned and confirmed to be a single-copy gene. Phylogenetic analysis revealed that the newly cloned cDNA could be grouped within the family of fish HNF1α and the signature motifs for HNF1α, e.g., the dimerization domain for HNF1 dimer formation (45), POU and homeobox domains for DNA binding (46, 47) and transactivation domain for promoter activation (48), were also identified in the protein sequence of carp HNF1α. By *in silico* modeling, the 3D structures of dimerization domain, POU domain, and homeobox domain of carp HNF1α were found to be highly comparable with their human counterparts. Functional expression in αT3 cells also confirmed that HNF1α of carp origin could transactivate target promoter with HBE sites. Our findings, taken together, indicate that the newly cloned cDNA indeed encodes the bona fide HNF1α with bioactivity in grass carp.

HNF1α is a liver-enriched transcription factor involved in glucose and lipid metabolism (49) and its mutation can lead to MODY3 subtype of diabetes (50). In mammals, “extra-hepatic” expression of HNF1α has also been reported, e.g., in the stomach, pancreas, intestine, and kidney of the mice (51). In agreement with its expression in the liver, pancreas, and kidney, HNF1α knockout in mouse model not only can induce metabolic defects at the hepatic level but also alter insulin secretion and reduce renal glucose reabsorption (52). In fish models, HNF1α has been cloned in salmon (53) and zebrafish (54), and its tissue expression is highly comparable to that in mammals (53, 54). In zebrafish, genome-wide promoter analysis reveals the presence of binding sites for HNF1, HNF3, HNF4, and HNF6 in a large number of liver-specific/enriched genes, suggesting that HNF1α by working with other HNFs may form a “transcriptional network” regulating hepatic functions (55). In tilapia, HNF1α expression can also be detected in the gonad (56) and it raises the possibility that HNF1α may play a role in steroid production/gametogenesis in fish models (57). In our study with grass carp, PCR signals for HNF1α were found to be ubiquitously expressed with the highest level detected in the liver, to a lower extent in the intestine, pituitary, kidney and gills, and with low levels in the heart, spleen, muscle, and various brain areas. The high level of HNF1α expression in the liver is in agreement with its role as a liver-enriched transcription factor (49), while the HNF1α signals detected in the gut and kidney are also comparable to the previous studies in zebrafish (54). However, our findings of HNF1α expression in the gills and pituitary, together with the signals within the brain, have not been reported previously and may suggest a possible role of HNF1α in osmoregulation at the branchial level and neurotransmission/neuroendocrine functions in the brain-pituitary axis. In parallel study with LC/MS/MS, protein fragments originated from HNF1α were also identified in protein samples prepared from the carp liver. These results not only provide evidence for hepatic expression of HNF1α at the protein level but also support the idea that the transcript signals for HNF1α detected by RT-PCR can be properly translated into target protein at the tissue level in grass carp.

Pancreatic hormones, including insulin and glucagon, are known to play a key role in regulating hepatic functions mainly via the portal vascular link between the liver and pancreas (58). Apart from their metabolic effects, insulin and glucagon can also modify hepatic expression of IGF-I, the down-stream effector of GH, but their effects are highly variable among different studies. In rat hepatocytes, insulin could induce IGF-I expression by increasing its transcript stability without affecting its gene transcription (59). In other reports with the same model, insulin was found to have no effect on basal (60) but enhance GH-induced IGF-I mRNA expression (61), probably by up-regulation of GH receptor expression (62). Similar inconsistency has also been documented for the studies with glucagon. In this case, glucagon was shown to have stimulatory (9), inhibitory (10) or no effects on IGF-I expression/secretion (63) and the cause for the discrepancy is still unclear. In fish models, similar information for IGF-I regulation is restricted to a limited number of studies. For examples, in tilapia, insulin treatment could inhibit basal and did not alter GH-induced IGF-I gene expression at the hepatic level (64). In salmon hepatocytes, interestingly, glucagon had no effect on basal but suppressed GH stimulation on IGF-I mRNA level (15). In our study with carp hepatocytes, we have the novel findings that insulin not only could increase basal but also potentiate GH-induced IGF-I gene expression through protein:protein interaction between GH receptor and insulin receptor with concurrent enhancement of STAT_5_, ERK_1/2_, and Akt signaling (32). The same model was also used in our current study to examine the mechanisms for IGF-I regulation by glucagon in the carp liver. In this case, glucagon treatment was found to elevate IGF-I mRNA levels with parallel rises in HNF1α and CREB transcript expression in carp hepatocytes. During the process, rapid phosphorylation of CREB and elevations in protein expression of HNF1α and CREB were also noted. Given that (i) HBE and CRE sites are present in the proximal promoter of carp IGF-I gene, (ii) the HNF1α and CREB responses (both mRNA and protein) could occur prior to the rise in IGF-I signals after glucagon induction, and (iii) glucagon-induced IGF-I mRNA expression could be blocked by inhibition of gene transcription and protein translation, it raises the possibility that HNF1α and CREB gene expression followed by their protein translation and subsequent activation (for CREB) may contribute to glucagon-induced IGF-I gene transcription in the carp liver.

In mammals, glucagon receptor is known to be functionally coupled with the cAMP/PKA and PLC/IP_3_/PKC pathways (35, 65). Glucagon activation of MAPK (66) and PI3K/Akt cascades (67) has also been reported, which can be attributed to the signaling crosstalk by Epac, a guanine nucleotide exchange factor activated by cAMP (66, 68). In carp hepatocytes, increasing cAMP synthesis by AC activation (using forskolin) or treatment with cAMP analog (e.g., 8cpt-cAMP) could mimic glucagon stimulation of IGF-I, HNF1α, and CREB mRNA whereas the corresponding responses triggered by glucagon were totally negated by inactivating AC (by MDL12330A) or PKA (by H89). In parallel experiments, IGF-I, HNF1α, and CREB mRNA levels were also elevated by increasing functional levels of DAG (by DiC8) or direct activation of PKC (using TPA) but their corresponding stimulation induced by glucagon were found to be highly sensitive to PLC blockade (by edelfosine), IP_3_ receptor inactivation (by 2-APB) or inhibiting PKC activity (by GF109203X). These findings, as a whole, support the idea that glucagon-induced IGF-I, HNF1α, and CREB expression in the carp liver were mediated by the AC/cAMP/PKA and PLC/IP_3_/PKC pathways. Regarding the role of MAPK and PI3K/Akt cascades, their involvement in hepatic expression of IGF-I induced by GH has been reported in fish models, e.g., in rainbow trout (69) and grass carp (26). In our study with carp hepatocytes, glucagon-induced IGF-I and CREB but not HNF1α mRNA expression could be reduced/totally abolished by blocking MEK_1/2_ (by PD98059) or ERK_1/2_ (by SCH772984). However, inhibiting PI3K (by Ly294002), Akt (by API-2), and mTOR (by rapamycin) were found to ablate IGF-I and HNF1α transcript expression induced by glucagon but the corresponding rise in CREB signals was not affected. These findings imply that (i) the MEK_1/2_/ERK_1/2_ and PI3K/Akt/mTOR cascades were involved in IGF-I induction by glucagon in addition to the cAMP/PKA and PLC/IP_3_/PKC pathways, (ii) hepatic expression of HNF1α induced by glucagon was mediated by the PI3K/Akt/mTOR but not MEK_1/2_/ERK_1/2_ pathway, and (iii) the parallel response for CREB, in contrast, was mediated by MEK_1/2_/ERK_1/2_ pathway and the PI3K/Akt/mTOR cascade was not involved. Apparently, glucagon induction of IGF-I, HNF1α and CREB expression in the carp liver was mediated by overlapping and yet distinct post-receptor signaling mechanisms.

Since Epac is known to mediate cAMP crosstalk with the MAPK and PI3K/Akt pathways (40) and glucagon actions mediated by Epac, e.g., for ghrelin regulation (66) and glucose homeostasis (41), have also been reported, it is tempting to speculate that Epac may be involved in glucagon induction of IGF-I, HNF1α, and CREB expression in carp liver. However, the idea is not supported by our findings based on cAMP analogs with differential selectivity for PKA and Epac. In carp hepatocytes, unlike the PKA-specific 6Bnz-cAMP which could mimic the stimulatory actions of glucagon, the Epac-specific 8cpt-2Me-cAMP was found to have no effects on transcript expression of IGF-I, HNF1a, and CREB. Similar to the results of glucagon treatment, the stimulatory effects of 6Bnz-cAMP on IGF-I and CREB but not HNF1α mRNA expression were sensitive to the blockade of MEK_1/2_ (by PD98059) and ERK_1/2_ (using SCH772984). Meanwhile, inhibiting PI3K (by Ly294002), Akt (by APB-2), and mTOR (by rapamycin) were also effective in blocking transcript expression of IGF-I and HNF1α induced by 6Bnz-cAMP but with no effect on the corresponding responses for CREB. These findings suggest that (i) the MEK_1/2_/ERK_1/2_ and PI3K/Akt/mTOR cascades were acting downstream of PKA activation for IGF-I expression, (ii) functional coupling of PKA with the PI3K/Akt/mTOR pathway was involved in HNF1α expression, and (iii) the corresponding responses for CREB expression were mediated by PKA coupling with the MEK_1/2_/ERK_1/2_ cascades and the PI3K/Akt/mTOR pathway was not involved. Since PKC activation of MAPK and PI3K/Akt cascades has also been documented, e.g., in curcumin prevention of diabetic cardiomyopathy (70) and actin remodeling in chemotaxing ameboid cells (71), the possibility of PKC crosstalk with MAPK and PI3K/Akt cascades in the hepatic actions of glucagon cannot be excluded. In carp hepatocytes, PKC activation (by TPA) consistently induced transcript expression of IGF-I, HNF1α, and CREB and the stimulatory actions on IGF-I and CREB but not HNF1α mRNA could be blocked by MEK_1/2_ (using PD98059) or ERK_1/2_ inactivation (by SCH772984). Parallel inhibition of PI3K (by Ly294002), Akt (by API-2), or mTOR (by rapamycin), however, were found to have no effects on the stimulatory responses induced by PKC activation. Our results indicate that, unlike PKA coupling with both MARK and PI3K/Akt cascades, the MEK_1/2_/ERK_1/2_ pathway was acting downstream of PKC and involved in IGF-I and CREB but not HNF1α gene expression. Although our studies have provided evidence for differential crosstalk of PKA and PKC with the MARK and PI3K/Akt cascades, we do not exclude the possibility that direct stimulation of MARK and PI3K/Akt signaling can also be achieved by glucagon receptor activation, e.g., by signal transduction via Gβγ protein (72).

In previous reports with mammalian models (e.g., rat), the studies on IGF-I regulation by glucagon have been focused on IGF-I secretion/transcript expression (9, 10) and no information is available for glucagon regulation of IGF-I gene transcription. In carp hepatocytes, glucagon treatment was found to enhance the recruitment of Pol-II to IGF-I promoter with parallel elevation in IGF-I primary transcript. Since Pol-II recruitment at promoter level (42) followed by primary transcript production (43) are the crucial steps for gene transcription, our findings may imply that the responses for IGF-I mRNA were caused by glucagon induction of IGF-I gene transcription. This idea is also supported by the results of transfection studies in αT3 cells with Luc reporters carrying the carp IGF-I promoter. In this case, IGF-I promoter activity could be elevated by glucagon treatment, and similar to carp hepatocytes, the trans-activating effect of glucagon on IGF-I promoter was mediated by cAMP/PKA, PLC/IP_3_/PKC, MAPK, and PI3K/Akt cascades as revealed by pharmacological blockade of different components of respective signaling pathways. Using 5′ deletion, the region of IGF-I promoter responsive to glucagon induction was mapped to the proximal promoter downstream of position −112 containing a CRE site sitting right next to a HBE site. Although the presence of CRE site in IGF-I promoter has not be reported previously, the HBE site located in IGF-I promoter of fish species, e.g., in salmon (24) and common carp (25), is well-conserved and known to be involved in GH-induced IGF-I promoter activity. In our study with carp hepatocytes, interestingly, we have the novel finding that glucagon treatment could induce rapid association of HNF1α with phosphorylated CREB. For parallel transfection studies in αT3 cells, over-expression of HNF1α or CREB increased both basal and glucagon-induced IGF-I promoter activity and these stimulatory actions could be further enhanced by co-expression of the two transcription factors. Of note, the enhancing effects on glucagon induction caused by HNF1α or CREB over-expression could be blocked by site-directed mutagenesis/truncation of the respective cis-acting elements within the IGF-I promoter. Our findings, as a whole, suggest that HNF1α and CREB expression (accompanied with CREB activation) induced by glucagon followed by their protein:protein interaction and transactivation via the CRE and HBE sites within the proximal region of IGF-I promoter can lead to Pol-II recruitment and IGF-I gene transcription in the carp liver.

**Figure 15:**
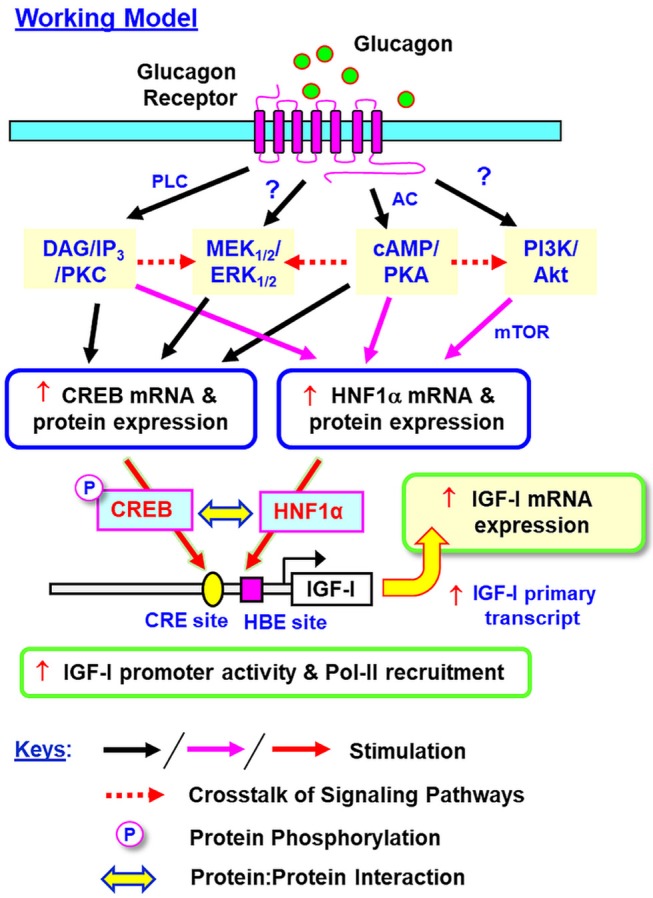
Working model for glucagon-induced IGF-I expression in the carp liver. In carp hepatocytes, glucagon can induce IGF-I gene expression via activation of the cAMP/PKA, PLC/IP_3_/PKC, MEK_1/2_/ERK_1/2_, and PI3K/Akt pathways. During the process, parallel rises in HNF1α and CREB expression (at both mRNA and protein levels) and protein phosphorylation of CREB can also be noted. Apparently, the cAMP/PKA and PLC/IP_3_/PKC pathways together with PKA crosstalk with the PI3K/Akt cascades are involved in glucagon-induced HNF1α expression. Interestingly, the corresponding responses for CREB are mediated by cAMP/PKA and PLC/IP_3_/PKC pathways together with the functional crosstalk of PKA and PKC with the MEK_1/2_/ERK_1/2_ cascade. Protein:protein interaction of HNF1α with phosphorylated CREB and transactivation via the CRE and HBE sites within the proximal promoter of IGF-I gene can trigger Pol-II recruitment and IGF-I gene transcription to initiate the subsequent production of IGF-I primary transcript and mature RNA. In this model, whether a direct induction of MEK_1/2_/ERK_1/2_ and PI3K/Akt cascades can also occur with glucagon receptor activation is still unclear and the possibility of “receptor coupling” via Gβγ cannot be excluded.

In summary, grass carp HNF1α was cloned and confirmed to be a single-copy gene expressed in the liver with the ability to transactivate target promoter with HBE sites. Based on our studies in grass carp hepatocytes and αT3 cells, a working model has been proposed for the signal transduction and transcriptional mechanisms mediating glucagon-induced IGF-I gene expression in the carp liver (Figure 15). In grass carp, glucagon can trigger IGF-I mRNA expression at the hepatic level via activation of the cAMP/PKA, PLC/IP_3_/PKC, MAPK, and PI3K/Akt cascades with parallel rises of HNF1α and CREB expression and CREB phosphorylation. The cAMP/PKA and PLC/IP_3_/PKC pathways activated by glucagon together with PKA crosstalk with PI3K/Akt/mTOR cascades are involved in the stimulatory effect on HNF1α expression. The corresponding actions on CREB expression, however, are mediated by glucagon activation of cAMP/PKA and PLC/IP_3_/PKC cascades with PKA and PKC crosstalk with the MEK_1/2_/ERK_1/2_ pathway. During glucagon stimulation, protein:protein interaction between HNF1α and phosphorylated CREB and transactivation of IGF-I promoter via the CRE and HBE sites within the proximal promoter region also occur, which then trigger RNA Pol-II recruitment and initiation of IGF-I gene transcription for subsequent production of IGF-I primary transcript and mature mRNA. Our studies for the first time provide information on (i) the post-receptor signaling mechanisms for glucagon-induced IGF-I gene expression at the hepatic level, and (ii) the novel responses of HNF1α and CREB expression induced by glucagon and their functional role in glucagon-induced IGF-I promoter activation. In mammals, Pol-II binding to IGF-I promoter (e.g., rat) with p300-dependent histone hyperacetylation (20) and HNF6 association with Foxa2 (73) or C/EBPα (74) to enhance promoter activity via p300/CBP recruitment have been reported (e.g., in HepG2 cells). In different cell models, the histone acetylases p300/CBP are known to bind with CREB and act as the co-activators for its transactivation activity via complex formation with CRTC2 (27). Whether p300/CBP recruitment can occur during HNF1α/CREB interaction induced by glucagon and contribute to IGF-I gene transcription by chromatin acetylation is unclear, and for sure, can be as an interesting direction for our on-going research.

## Data Availability

The datasets generated for this study can be found in the GenBank.

## Ethics Statement

This study was carried out in accordance with the recommended guidelines for the care and use of animals for research and teaching at the University of Hong Kong (Hong Kong). The protocol used in our study (CULATR4608-18) was approved by the Committee on the Use of Live Animal for Teaching and Research, University of Hong Kong.

## Author Contributions

AW was the PI and grant holder. AW and JB were responsible for project planning and data analysis. JB and XJ were involved in molecular cloning and functional studies for IGF-I, HNF1α, and CREB expression. MH was responsible for LC/MS/MS detection of HNF1α protein expression in the liver. Manuscript preparation and editing were done by AW, BC, and JB.

### Conflict of Interest Statement

The authors declare that the research was conducted in the absence of any commercial or financial relationships that could be construed as a potential conflict of interest.

## Abbreviations

IGF-I: Insulin-like growth factor I
GH: Growth hormone
HNF1α: Hepatic nuclear factor 1α
CREB: cAMP response element binding protein
HBE: HNF1α binding element
CRE: cAMP response element
RNA Pol-II: RNA polymerase II
ChIP: Chromatin immunoprecipitation
AC: Adenylyl cyclase
PKA: Protein kinase A
PLC: Phospholipase C
PKC: Protein kinase C
IP_3_: Inositol 1,4,5-triphosphate
DAG: Diacylgerol
MAPK: Mitogen-activated protein kinases
MEK_1/2_: Mitogen-activated protein kinase kinase 1/2
ERK_1/2_: Extracellular signal-regulated kinase 1/2
PI3K: Phosphoinositide 3-kinase
Akt: Protein kinase B
mTOR: Mammalian target of rapamycin.

## References

[1] Doi Y Iwai M Matsuura B Onji M. Glucagon attenuates the action of insulin on glucose output in the liver of the Goto-Kakizaki rat perfused *in situ*. Pflugers Arch. (2001) 442:537–41. 10.1007/s004240100573 11510886

[2] Ramnanan CJ Edgerton DS Kraft G Cherrington AD. Physiologic action of glucagon on liver glucose metabolism. Diabetes Obes Metab. (2011) 13(Suppl. 1):118–25. 10.1111/j.1463-1326.2011.01454.x 21824265 PMC5371022

[3] Jones JG. Hepatic glucose and lipid metabolism. Diabetologia. (2016) 59:1098–103. 10.1007/s00125-016-3940-5 27048250

[4] Holst JJ Wewer Albrechtsen NJ Pedersen J Knop FK. Glucagon and amino acids are linked in a mutual feedback cycle: the liver-α cell sxis. Diabetes. (2017) 66:235–40. 10.2337/db16-0994 28108603

[5] Muller TD Finan B Clemmensen C DiMarchi RD Tschop MH. The new biology and pharmacology of glucagon. Physiol Rev. (2017) 97:721–66. 10.1152/physrev.00025.2016 28275047

[6] Micmacher E Assumpcao RP Redorat RG Spina LD Cruz IC Silva CA. Growth hormone secretion in response to glucagon stimulation test in healthy middle-aged men. Arq Bras Endocrinol Metabol. (2009) 53:853–8. 10.1590/S0004-27302009000700009 19942987

[7] Cain JP Williams GH Dluhy RG. Glucagon-initiated human growth hormone release: a comparative study. Can Med Assoc J. (1972) 107:617–22. 4665093 PMC1940964

[8] Weber B Helge H Quabbe HJ. Glucagon-induced growth hormone release in children. Acta Endocrinol. (1970) 65:323–41. 10.1530/acta.0.0650323 5535989

[9] Kachra Z Barash I Yannopoulos C Khan MN Guyda HJ Posner BI. The differential regulation by glucagon and growth hormone of insulin-like growth factor (IGF)-I and IGF binding proteins in cultured rat hepatocytes. Endocrinology. (1991) 128:1723–30. 10.1210/endo-128-4-1723 1706258

[10] Denver RJ Nicoll CS. Pancreatic hormones differentially regulate insulin-like growth factor (IGF)-I and IGF-binding protein production by primary rat hepatocytes. J Endocrinol. (1994) 142:299–310. 10.1677/joe.0.1420299 7523561

[11] Authier F Desbuquois B. Glucagon receptors. Cell Mol Life Sci. (2008) 65:1880–99. 10.1007/s00018-008-7479-6 18292967 PMC11131795

[12] Sinclair EM Yusta B Streutker C Baggio LL Koehler J Charron MJ. Glucagon receptor signaling is essential for control of murine hepatocyte survival. Gastroenterology. (2008) 135:2096–106. 10.1053/j.gastro.2008.07.075 18809404

[13] Chow BK Moon TW Hoo RL Yeung CM Muller M Christos PJ. Identification and characterization of a glucagon receptor from the goldfish *Carassius auratus*: implications for the evolution of the ligand specificity of glucagon receptors in vertebrates. Endocrinology. (2004) 145: 3273–88. 10.1210/en.2003-0597 15033912

[14] Moon TW Hormones and fish hepatocyte metabolism: “The good, the bad and the ugly!”. Comp Biochem Physiol B Biochem Mol Biol. (2004) 139:335–45. 10.1016/j.cbpc.2004.06.003 15544959

[15] Pierce AL Fukada H Dickhoff WW. Metabolic hormones modulate the effect of growth hormone (GH) on insulin-like growth factor-I (IGF-I) mRNA level in primary culture of salmon hepatocytes. J Endocrinol. (2005) 184:341–9. 10.1677/joe.1.05892 15684342

[16] Chia DJ Ono M Woelfle J Schlesinger-Massart M Jiang H Rotwein P. Characterization of distinct Stat5b binding sites that mediate growth hormone-stimulated IGF-I gene transcription. J Biol Chem. (2006) 281:3190–7. 10.1074/jbc.M510204200 16339156

[17] Kitanaka S Sato U Igarashi T. Regulation of human insulin, IGF-I, and multidrug resistance protein 2 promoter activity by hepatocyte nuclear factor (HNF)-1β and HNF-1α and the abnormality of HNF-1β mutants. J Endocrinol. (2007) 192:141–7. 10.1677/joe.1.07003 17210751

[18] Eleswarapu S Ge X Wang Y Yu J Jiang H Growth hormone-activated STAT5 may indirectly stimulate IGF-I gene transcription through HNF-3γ. Mol Endocrinol. (2009) 23:2026–37. 10.1210/me.2009-0178 19819986 PMC2796150

[19] Staiger J Lueben MJ Berrigan D Malik R Perkins SN Hursting SD. C/EBPbeta regulates body composition, energy balance-related hormones and tumor growth. Carcinogenesis. (2009) 30: 832–40. 10.1093/carcin/bgn273 19056928 PMC2675647

[20] Chia DJ Young JJ Mertens AR Rotwein P. Distinct alterations in chromatin organization of the two IGF-I promoters precede growth hormone-induced activation of IGF-I gene transcription. Mol Endocrinol. (2010) 24:779–89. 10.1210/me.2009-0430 20160126 PMC2852351

[21] Rotwein P. Mapping the growth hormone/Stat5b/IGF-I transcriptional circuit. Trends Endocrinol Metab. (2012) 23:186–93. 10.1016/j.tem.2012.01.001 22361342 PMC3313013

[22] Rotwein P Insulin-like growth factor 1 gene variation in vertebrates. Endocrinology. (2018) 159: 2288–305. 10.1210/en.2018-00259 29697760 PMC6692883

[23] Kulik VP Kavsan VM van Schaik FM Nolten LA Steenbergh PH Sussenbach JS. The promoter of the salmon insulin-like growth factor I gene is activated by hepatocyte nuclear factor 1. J Biol Chem. (1995) 270:1068–73. 10.1074/jbc.270.3.1068 7836361

[24] Meton I Boot EP Sussenbach JS Steenbergh PH. Growth hormone induces insulin-like growth factor-I gene transcription by a synergistic action of STAT5 and HNF-1α. FEBS Lett. (1999) 444:155–9. 10.1016/S0014-5793(99)00064-2 10050749

[25] Vong QP Chan KM Leung K Cheng CH. Common carp insulin-like growth factor-I gene: complete nucleotide sequence and functional characterization of the 5'-flanking region. Gene. (2003) 322:145–56. 10.1016/j.gene.2003.08.019 14644506

[26] Jiang X Xiao J He M Ma A Wong AO Type II SOCS as a feedback repressor for GH-induced *Igf1* gene expression in carp hepatocytes. J Endocrinol. (2016) 229:171–86. 10.1530/JOE-15-0423 27271287

[27] Altarejos JY Montminy M. CREB and the CRTC co-activators: sensors for hormonal and metabolic signals. Nat Rev Mol Cell Biol. (2011) 12:141–51. 10.1038/nrm3072 21346730 PMC4324555

[28] Sun X Dang F Zhang D Yuan Y Zhang C Wu Y. Glucagon-CREB/CRTC2 signaling cascade regulates hepatic BMAL1 protein. J Biol Chem. (2015) 290:2189–97. 10.1074/jbc.M114.612358 25480789 PMC4303670

[29] Yan A Chen T Chen S Tang D Liu F Jiang X. Signal transduction mechanism for glucagon-induced leptin gene expression in goldfish liver. Int J Biol Sci. (2016) 1544–54. 10.7150/ijbs.16612 27994518 PMC5166495

[30] Huo L Fu G Wang X Ko WK Wong AO. Modulation of calmodulin gene expression as a novel mechanism for growth hormone feedback control by insulin-like growth factor in grass carp pituitary cells. Endocrinology. (2005) 146:3821–35. 10.1210/en.2004-1508 15932934

[31] Wong MK Sze KH Chen T Cho CK Law HC Chu IK. Goldfish spexin: solution structure and novel function as a satiety factor in feeding control. Am J Physiol Endocrinol Metab. (2013) 305: E348–66. 10.1152/ajpendo.00141.2013 23715729

[32] Jiang Q Bai J He M Yuen KW Wong AO. Mechanisms underlying the synergistic action of insulin and growth hormone on IGF-I and -II expression in grass carp hepatocytes. Front Endocrinol. (2018) 9:336. 10.3389/fendo.2018.00336 29977227 PMC6021495

[33] Bai J Gong W Wang C Gao Y Hong W Chen SX. Dynamic methylation pattern of *cyp19a1a* core promoter during zebrafish ovarian folliculogenesis. Fish Physiol Biochem. (2016) 42:947–54. 10.1007/s10695-015-0187-x 26719066

[34] Lin C Bai J He M Wong AO. Grass carp prolactin gene: Structural characterization and signal transduction for PACAP-induced prolactin promoter activity. Sci Rep. (2018), 8:4655. 10.1038/s41598-018-23092-0 29545542 PMC5854708

[35] Rodgers RL. Glucagon and cyclic AMP: time to turn the page? Curr Diabetes Rev. (2012) 8:362–81. 10.2174/157339912802083540 22587514

[36] Galsgaard KD Pedersen J Knop FK Holst JJ Wewer Albrechtsen NJ. Glucagon receptor signaling and lipid metabolism. Front Physiol. (2019) 10:413. 10.3389/fphys.2019.00413 31068828 PMC6491692

[37] Dalle S Longuet C Costes S Broca C Faruque O Fontes G. Glucagon promotes cAMP-response element-binding protein phosphorylation via activation of ERK_1/2_ in MIN6 cell line and isolated islets of Langerhans. J Biol Chem. (2004) 279:20345–55. 10.1074/jbc.M312483200 14988413

[38] Li XC Carretero OA Shao Y Zhuo JL. Glucagon receptor-mediated extracellular signal-regulated kinase 1/2 phosphorylation in rat mesangial cells: role of protein kinase A and phospholipase C. Hypertension. (2006) 47:580–5. 10.1161/01.HYP.0000197946.81754.0a 16391176 PMC2367309

[39] Shen B Kwan HY Ma X Wong CO Du J Huang Y. cAMP activates TRPC6 channels via the phosphatidylinositol 3-kinase (PI3K)-protein kinase B (PKB)-mitogen-activated protein kinase kinase (MEK)-ERK_1/2_ signaling pathway. J Biol Chem. (2011) 286:19439–45. 10.1074/jbc.M110.210294 21487005 PMC3103323

[40] Chen H Wild C Zhou X Ye N Cheng X Zhou J. Recent advances in the discovery of small molecules targeting exchange proteins directly activated by cAMP (EPAC). J Med Chem. 2014, 57: 3651–65. 10.1021/jm401425e 24256330 PMC4016168

[41] Almahariq M Mei FC Cheng X. Cyclic AMP sensor EPAC proteins and energy homeostasis. Trends Endocrinol Metab. (2014) 25:60–71. 10.1016/j.tem.2013.10.004 24231725 PMC3946731

[42] Hahn S. Structure and mechanism of the RNA polymerase II transcription machinery. Nat Struct Mol Biol. (2004) 11:394–403. 10.1038/nsmb763 15114340 PMC1189732

[43] Beyersmann D. Regulation of mammalian gene expression. EXS. (2000) 89:11–28. 10.1007/978-3-0348-8393-1_2 10997280

[44] Nolten LA Steenbergh PH Sussenbach JS Hepatocyte nuclear factor 1α activates promoter 1 of the human insulin-like growth factor I gene via two distinct binding sites. Mol Endocrinol. (1995) 9:1488–99. 10.1210/me.9.11.1488 8584026

[45] Locker J Ghosh D Luc PV Zheng J. Definition and prediction of the full range of transcription factor binding sites - the hepatocyte nuclear factor 1 dimeric site. Nucleic Acids Res. (2002) 30:3809–17. 10.1093/nar/gkf484 12202766 PMC137408

[46] Burglin TR Affolter M. Homeodomain proteins: an update. Chromosoma. (2016) 125:497–521. 10.1007/s00412-015-0543-8 26464018 PMC4901127

[47] Sneha P Thirumal KD George PD Siva R Zayed H Determining the role of missense mutations in the POU domain of HNF1A that reduce the DNA-binding affinity: a computational approach. PLoS ONE. (2017) 12:e0174953 10.1371/journal.pone.0174953 28410371 PMC5391926

[48] Mendel DB Crabtree GR. HNF-1, a member of a novel class of dimerizing homeodomain proteins. J Biol Chem. (1991) 266:677–80. 1985954

[49] Lau HH Ng NH Loo LS Jasmen JB Teo AK. The molecular functions of hepatocyte nuclear factors–in and beyond the liver. J Hepatol. (2018) 68:1033–48. 10.1016/j.jhep.2017.11.026 29175243

[50] Kulkarni RN Kahn CR. Molecular biology of HNFs: linking the liver and pancreatic islets in diabetes. Science. (2004) 303:1311–2. 10.1126/science.1095486 14988544

[51] Kuo CJ Conley PB Hsieh CL Francke U Crabtree GR. Molecular cloning, functional expression, and chromosomal localization of mouse hepatocyte nuclear factor 1. Proc Natl Acad Sci USA. (1990) 87:9838–42. 10.1073/pnas.87.24.9838 2263635 PMC55269

[52] Pontoglio M Prie D Cheret C Doyen A Leroy C Froguel P. HNF1α controls renal glucose reabsorption in mouse and man. EMBO Rep. (2000) 1:359–65. 10.1093/embo-reports/kvd071 11269503 PMC1083745

[53] Deryckere F Byrnes L Wagner A McMorrow T Gannon F. Salmon HNF1: cDNA sequence, evolution, tissue specificity and binding to the salmon serum albumin promoter. J Mol Biol. (1995) 247:1–10. 10.1006/jmbi.1994.0115 7897653

[54] Gong HY Lin CJ Chen MH Hu MC Lin GH Zhou Y. Two distinct teleost hepatocyte nuclear factor 1 genes, *hnf1*α*/tcf1* and *hnf1*β*/tcf2*, abundantly expressed in liver, pancreas, gut and kidney of zebrafish. Gene. (2004) 338:35–46. 10.1016/j.gene.2004.05.003 15302404

[55] Cheng W Guo L Zhang Z Soo HM Wen C Wu W. HNF factors form a network to regulate liver-enriched genes in zebrafish. Dev Biol. (2006) 294:482–96. 10.1016/j.ydbio.2006.03.018 16631158

[56] Huang WT Gong HY Lin CJ Weng CF Chen MH Wu JL. Hepatocyte nuclear factors-1α,−1β, and−3β expressed in the gonad of tilapia (*Oreochromis mossambicus*). Biochem Biophys Res Commun. (2001) 288:833–40. 10.1006/bbrc.2001.5856 11688983

[57] Huang WT Weng CF. Roles of hepatocyte nuclear factors (HNF) in the regulation of reproduction in teleosts. J Fish Biol. (2010) 76:225–39. 10.1111/j.1095-8649.2009.02480.x 20738706

[58] Griffen SC Russell SM Katz LS Nicoll CS. Insulin exerts metabolic and growth-promoting effects by a direct action on the liver *in vivo*: clarification of the functional significance of the portal vascular link between the beta cells of the pancreatic islets and the liver. Proc Natl Acad Sci USA. (1987) 84:7300–4. 10.1073/pnas.84.20.7300 3313390 PMC299280

[59] Goya L de la Puente A Ramos S Martin MA Escriva F Alvarez C. Regulation of IGF-I and -II by insulin in primary cultures of fetal rat hepatocytes. Endocrinology. (2001) 142:5089–96. 10.1210/endo.142.12.8521 11713201

[60] Johnson TR Blossey BK Denko CW Ilan J. Expression of insulin-like growth factor I in cultured rat hepatocytes: effects of insulin and growth hormone. Mol Endocrinol. (1989) 3:580–7. 10.1210/mend-3-3-580 2664476

[61] Boni-Schnetzler M Schmid C Meier PJ Froesch ER. Insulin regulates insulin-like growth factor I mRNA in rat hepatocytes. Am J Physiol. (1991) 260:E846–51. 10.1152/ajpendo.1991.260.6.E846 2058660

[62] Leung KC Doyle N Ballesteros M Waters MJ Ho KK. Insulin regulation of human hepatic growth hormone receptors: divergent effects on biosynthesis and surface translocation. J Clin Endocrinol Metab. (2000) 85:4712–20. 10.1210/jc.85.12.4712 11134133

[63] Sarem Z Bumke-Vogt C Mahmoud AM Assefa B Weickert MO Adamidou A: Glucagon decreases IGF-I bioactivity in humans, independently of insulin, by modulating its binding proteins. J Clin Endocrinol Metab. (2017) 102:3480–90. 10.1210/jc.2017-00558 28911141 PMC6287397

[64] Pierce AL Breves JP Moriyama S Hirano T Grau EG. Differential regulation of *Igf1* and *Igf2* mRNA levels in tilapia hepatocytes: effects of insulin and cortisol on GH sensitivity. J Endocrinol. (2011) 211:201–10. 10.1530/JOE-10-0456 21803836

[65] Mayo KE Miller LJ Bataille D Dalle S Goke B Thorens B. International union of pharmacology. XXXV. The glucagon receptor family. Pharmacol Rev. (2003) 55:167–94. 10.1124/pr.55.1.6 12615957

[66] Gagnon J Anini Y. Glucagon stimulates ghrelin secretion through the activation of MAPK and EPAC and potentiates the effect of norepinephrine. Endocrinology. (2013) 154:666–74. 10.1210/en.2012-1994 23307791

[67] Soria LR Gradilone SA Larocca MC Marinelli RA Glucagon induces the gene expression of aquaporin-8 but not that of aquaporin-9 water channels in the rat hepatocyte. Am J Physiol Regul Integr Comp Physiol. (2009) 296:R1274–81. 10.1152/ajpregu.90783.2008 19193945

[68] Khouri C Dittrich A Sackett SD Denecke B Trautwein C Schaper F. Glucagon counteracts interleukin-6-dependent gene expression by redundant action of Epac and PKA. Biol Chem. (2011) 392:1123–34. 10.1515/BC.2011.171 22050227

[69] Reindl KM Kittilson JD Bergan HE Sheridan MA. Growth hormone-stimulated insulin-like growth factor-1 expression in rainbow trout (*Oncorhynchus mykiss*) hepatocytes is mediated by ERK, PI3K-AKT, and JAK-STAT. Am J Physiol Regul Integr Comp Physiol. (2011) 301:R236–43. 10.1152/ajpregu.00414.2010 21490369

[70] Soetikno V Sari FR Sukumaran V Lakshmanan AP Mito S Harima M. Curcumin prevents diabetic cardiomyopathy in streptozotocin-induced diabetic rats: possible involvement of PKC-MAPK signaling pathway. Eur J Pharm Sci. (2012) 47:604–14. 10.1016/j.ejps.2012.04.018 22564708

[71] Ziemba BP Burke JE Masson G Williams RL Falke JJ. Regulation of PI3K by PKC and MARCKS: Single-molecule analysis of a reconstituted signaling pathway. Biophys J. (2016) 110:1811–25. 10.1016/j.bpj.2016.03.001 27119641 PMC4850241

[72] Senarath K Kankanamge D Samaradivakara S Ratnayake K Tennakoon M Karunarathne A. Regulation of G protein β*γ* signaling. Int Rev Cell Mol Biol. (2018) 339:133–91. 10.1016/bs.ircmb.2018.02.008 29776603

[73] Rausa FM Tan Y Costa RH. Association between hepatocyte nuclear factor 6 (HNF-6) and FoxA2 DNA binding domains stimulates FoxA2 transcriptional activity but inhibits HNF-6 DNA binding. Mol Cell Biol. (2003) 23:437–49. 10.1128/MCB.23.2.437-449.2003 12509444 PMC151533

[74] Yoshida Y Hughes DE Rausa FM Kim IM Tan Y Darlington GJ. C/EBPalpha and HNF6 protein complex formation stimulates HNF6-dependent transcription by CBP coactivator recruitment in HepG2 cells. Hepatology. (2006) 43:276–86. 10.1002/hep.21044 16440369 PMC1360165

