# Supplementary material for: Novel Mechanisms for IGF-I Regulation by Glucagon in Carp Hepatocytes: Up-Regulation of HNF1α and CREB Expression via Signaling Crosstalk for IGF-I Gene Transcription
Source: Front Endocrinol (Lausanne). 2019 Sep 3;10:605. doi: 10.3389/fendo.2019.00605 (PMC6734168; doi:10.3389/fendo.2019.00605)
Supplement: Supplementary file 1 [file Data_Sheet_1.PDF]

Supplemental Table.1. Primer sequences and PCR conditions for real time PCR

Gene Target / GenBank Accession No	PCR Condition					Product Size
Sequences of forward & reverse primers	Denature	Anneal	Extension	Detection	Cycle No	& *Tm* value
IGF-I proximal promoter / KF199853.1						
F: 5’-TGACAGGGTGCCCAAAATCCTTAAT-3’	94 °C	53°C	72 °C	76 °C	×30	185 bp &
R: 5’-GACTTTGTTCCATTGCGCAGGC-3’	30 sec	30 sec	30 sec	20 sec		86.8 °C
IGF-I primary transcript / KF199853.1						
F: 5’-GCCTCGAGATGTACTGTGCACCC-3’	94 °C	53°C	72 °C	79°C	×40	337 bp &
R: 5’-TGCAGGGGAACACAATCAGTA-3’	30 sec	30 sec	30 sec	20 sec		86.1 °C
IGF-I mRNA / EU051323.1						
F: 5’-TCTCACTGGTGCTGTGCGTCCTCGCG-3’	94 °C	55 °C	72 °C	84 °C	×40	203 bp &
R: 5’-GCTCTGAAAGCAGCATTCGTCCACAA-3’	30 sec	30 sec	30 sec	20 sec		87.5 °C
HNF1α mRNA / MH751508.1						
F: 5’-ATGGCAACAATGGCACAGT-3’	94 °C	58 °C	72 °C	78 °C	×40	235 bp &
R: 5’-ACGTGCCACCCGATCCAGACTTCC-3’	30 sec	30 sec	30 sec	20 sec		84.2 °C
CREB mRNA / EF437961.1						
F: 5’-TCAACCTTCAGTCATTCAGTCA-3’	94 °C	56 °C	72 °C	85°C	×40	231 bp &
R: 5’-GTTGGCACTGTCACTGTAGTGA-3’	30 sec	30 sec	30 sec	20 sec		84.9 °C
18S RNA / HQ615531.1						
F: 5’-AGCAACTTTAGTATACGCTATT-3’	94 °C	57 °C	72 °C	79 °C	×40	210 bp &
R: 5’-CTGAGAAACGGCTACCACATC-3’	30 sec	30 sec	30 sec	20 sec		85.0 °C
