# Supplementary material for: Novel Mechanisms for IGF-I Regulation by Glucagon in Carp Hepatocytes: Up-Regulation of HNF1α and CREB Expression via Signaling Crosstalk for IGF-I Gene Transcription
Source: Front Endocrinol (Lausanne). 2019 Sep 3;10:605. doi: 10.3389/fendo.2019.00605 (PMC6734168; doi:10.3389/fendo.2019.00605)
Supplement: Supplementary file 2 [file Data_Sheet_2.PDF]

Supplemental Fig.1

1 M E G G E G R R A G G E R S S R L S A L Q E Q L V W S L L

88 GGC TCG GGA CTG TCA AAA GAG CTG CTG ATT CAG GCC ATG GGA GAT CTG GAG CGA GAA CGG GCC TCA ACC GGT GCC GAG AGG ACG GAC

30 G S G L S K E L L I Q A M G D L E R E R A S T G A E R T D

175 CGG GCC GAC GGC GAG AGC TCG GAG GAG GGA GAG ATG GAA AAT CCT CCT CCC ATC TTC CAT GAT CTG GAG AGG CTT CCG CCG GAG GAG

59 R A D G E S S E E G E M E N P P P I F H D L E R L P P E E

262 GCC GCG AGG CAA AGG GCT GAA GTC GAC CAG CTG TTG CAA GAG GAT CCC TGG CAT GTG GCT AAA ATA GTG AAG AGT TAT ATG CAG CAG

88 A A R Q R A E V D Q L L Q E D P W H V A K I V K S Y M Q Q

349 CAC AAC CTA CCA CAG AGA GAG GTG GTG GAG TCC ACC GGC CTC AAC CAA TCC CAT CTC TCC CAG CAC CTC AAC AAG GGC ACA CCC ATG

117 H N L P Q R E V V E S T G L N Q S H L S Q H L N K G T P M

436 AAG AAT CAA AAG CGT GCC GCC CTG TAC AGC TGG TAC ATC AAG AAA CAG ACA GAG ATC AGT CAA CAA TTC ACC AAT GCC AGT CGA GGT

146 K N Q K R A A L Y S W Y I K K Q T E I S Q Q F T N A S R G

523 GTC ATG TCA GGA GAG GAA TCC GGG GAG GAT GTG AGA AAG GGA CGG AGG AAT CGA TTC AAA TGG GGC CCC GCC TCC CAG CAA ATC TTG

175 V M S G E E S G E D V R K G R R N R F K W G P A S Q Q I L

610 TTC CAG GCT TAT GAA CGA CAG AAG AAC CCC AGT AAG GAG GAG AGG GAG GGA CTG GTG GAA GAG TGC AAC AGA GCG GAG TGT CTT CAG

204 F Q A Y E R Q K N P S K E E R E G L V E E C N R A E C L Q

697 AGG GGA GTT TCT CCT TCT CAG CTG GCT GGC CTG GGC TCC AAT TTG GTC ACA GAG GTT CGT GTA TAT AAT TGG TTT GCG AAT CGG CGT

233 R G V S P S Q L A G L G S N L V T E V R V Y N W F A N R R

784 AAA GAG GAG GCC TTT CGC CAC AAA CTG GCA CTG GAT GTG CCC TAC AGC AGC CAA ACT GCT TCC TCC ACA GGA CAG ACA CTA CCA TCT

262 K E E A F R H K L A L D V P Y S S Q T A S S T G Q T L P S

871 AGT CCT TCA CCA GGT CTG AAG TAC AGC CAG TCA GTG CTG TGT GAG AGT TTG GGC ACA ATG AGA AGC TCC AGT GGT GAA GGC AGG GCT

291 S P S P G L K Y S Q S V L C E S L G T M R S S S G E G R A

958 GGC AGC GGT CGT CTA TCC AGT CCA GTT CAG CTA GAA CCT AGT CAT ACA CTC CTG GAC ACA CAC CAT CAC AAA TCA GTA CCT GGT GGT

320 G S G R L S S P V Q L E P S H T L L D T H H H K S V P G G

1045 GGC TCC TTA CCT CCT GTC AGT ACT CTC ACT TCC CTG CAC GGA GTG TCT GGA TCC TCC GCT GGC CCT CCA GGA CTG ATC ATG GCC TCC

349 G S L P P V S T L T S L H G V S G S S A G P P G L I M A S

1132 CTA CCA AGT GTA ATG AGT CTG GGA GAC TCC TCA CTT CTC ATA GGT TTG ACG TCC TCT CAA CCA CAG ACG GTC CCT GTC ATC AAC AAC

378 L P S V M S L G D S S L L I G L T S S Q P Q T V P V I N N

1219 ATG GGG GGA GGG TTT ACT ACC CTT CAG CCA ATC TCG TTT CAG CAG CAG CTT CAA GCC TCA CCT CAG CAG CCA ATA GCA CAG CAG CTC

407 M G G G F T T L Q P I S F Q Q Q L Q A S P Q Q P I A Q Q L

1306 CAG TCT CAC ATT AGC CCA AGC TCC TTC ATG GCA ACA ATG GCA CAG TTC CCA TGC CAC ATG TAT AGC AAA GCA GAC CTG AGC TCT TAC

436 Q S H I S P S S F M A T M A Q F P C H M Y S K A D L S S Y

1393 CCC TCC TCC AGT CTC CTG TCC CAG GCT ATG GTC ATC GCA GAC AGC AAC AGC ATT GGG ACA CTG ACT AAC CTC ACA GCT GTT CGA CAG

465 P S S S L L S Q A M V I A D S N S I G T L T N L T A V R Q

1480 ATT CTC ACC TCA GAT CCT GAG GGT CAC ACA GAC ACT CCC ATT GAA GAG GAG TCT TTA CAT CTG CAA TCC ACT TCA CCA GAA CCA GCA

494 I L T S D P E G H T D T P I E E E S L H L Q S T S P E P A

1567 TCT TCG GGT AGT TTG GAG CTC TAC CCT CAG ACC CAG ACA AGT GAA AGC CAT CCT TCA CAC CTG CTC TCC TCT TCA CCA GGG GAC ATC

523 S S G S L E L Y P Q T Q T S E S H P S H L L S S S P G D I

1654 GAC CCT TAC ATC CCT ACA CAG ATG GTC TCT ACT GCA CAA TAG

552 D P Y I P T Q M V S T A Q *

Supplemental Fig.1. Nucleotide and amino acid (a.a.) sequences of grass carp HNF1α. The full-length cDNA of carp HNF1α is composed of 1695 bp encoding a 564 a.a. protein (with deduced MW of ~63 kDa). Within the coding region, the dimerization domain in the N-terminal is marked by dotted line and POU domain is shaded in grey. The homeobox domain is boxed and transactivation domain in the C-terminal is underlined for recognition. An asterisk (*) represents the stop codon located at the end of the ORF sequence.
