# Supplementary material for: Novel Mechanisms for IGF-I Regulation by Glucagon in Carp Hepatocytes: Up-Regulation of HNF1α and CREB Expression via Signaling Crosstalk for IGF-I Gene Transcription
Source: Front Endocrinol (Lausanne). 2019 Sep 3;10:605. doi: 10.3389/fendo.2019.00605 (PMC6734168; doi:10.3389/fendo.2019.00605)
Supplement: Supplementary file 3 [file Data_Sheet_3.PDF]

Supplemental Fig.2

```
                Dimerization domain
Grass carp  1   MEGGEGRRAGGERSSRLSALQEQLVWSLLGSGLSKELLIQAMGD------------------LERERAST-----------GAERTDRAD 61
Zebrafish   1   MDGGESRRSEG--SGRLSALQEQLVWSLLGSGLSKELLIQAMGD------------------LERERASS-----------GGERADRAD 59
Tilapia     1   MEGEERVEAAKAGPSRLTALQEQLIWALLESGLSREVLIQAMGE------------------LERNKASA-----------GNERGERGD 61
Xenopus     1   ------------MASQLSYLQRELLRALLESGVTKEALKKALADGEDYTYPNVPLDDIRN--LDEGENCV-------QLPNGLGDPQISE 69
Chicken     1   ------------MVSKLSHLQVELLGALLESGLTKETLIKALSEAEPYVLQSESQHAINALQTEKGESCP----EIPNLPNGMGETRLSE 74
Alligator   1   ------------MVAKLSHLQVELLGALLESGLTKETLIKALGEVDPYPLQSESQRAISAIQGEKGEPCA----EIPNLPNGMGESRLSE 74
Mouse       1   ------------MVSKLSQLQTELLAALLESGLSKEALIQALGEPGPYLMVGEGP-------LDKGESCGGSRGDLTELPNGLGETRGSE 71
Human       1   ------------MVSKLSQLQTELLAALLESGLSKEALIQALGEPGPYLLAGEGP-------LDKGESCGGGRGELAELPNGLGETRGSE 71

                                                           POU domain
Grass carp  62  GESSEEGEMENPPPIFHDLERLPPEEAARQRAEVDQLLQEDPWHVAKIVKSYMQQHNLPQREVVESTGLNQSHLSQHLNKGTPMKNQKRA 151
Zebrafish   60  CESSEEGEMDNPPPIYRELEKLPPEEAARQRAQVDQLLQEDPWHVAKMVKSYMQQHNLPQREVVESTGLNQSHLSQHLNKGTPMKNQKRA 149
Tilapia     62  GESSEEGEMDFPPPIFRELEKLPPEEASKLRTEVDHLLHEDPWHVAKMVKSYMQQHNLPQREVVESTGLNQSHLSQHLNKGTPMKNQKRA 151
Xenopus     70  DESSDDG-GDFTPPIMKELERLSPEEAAHQKAVVERLLQEDPWHVAKLIKSYLQQHNIPQREVVDTTGLNQSHLSQHLNKGTPMKTQKRA 158
Chicken     75  DETSDDG-EEFTPPIMKELENLSPEEAAHQKAVVERLLQEDPWRVAKMVKSYLQQHNIPQREVVDTTGLNQSHLSQHLNKGTPMKTQKRA 163
Alligator   75  EETSDDG-EEFTPPIMKELENLSPEEAAHQKAVVERLLQEDPWRVAKMVKSYLQQHNIPQREVVDTTGLNQSHLSQHLNKGTPMKTQKRA 163
Mouse       72  DDTDDDG-EDFAPPILKELENLSPEEAAHQKAVVESLLQEDPWRVAKMVKSYLQQHNIPQREVVDTTGLNQSHLSQHLNKGTPMKTQKRA 160
Human       72  DETDDDG-EDFTPPILKELENLSPEEAAHQKAVVETLLQEDPWRVAKMVKSYLQQHNIPQREVVDTTGLNQSHLSQHLNKGTPMKTQKRA 160

                                                         Homeobox domain
Grass carp  152 ALYSWYIKKQTEISQQFTNASRGVMSGEESGEDV--RKGRRNRFKWGPASQQILFQAYERQKNPSKEEREGLVEECNRAECLQRGVSPSQ 239
Zebrafish   150 ALYSWYTKKQAEISQQFTNASRGVMSGEEPGEDV--RKGRRNRFKWGPASLQILFQAYERQKNPSKEEREGLVEECNRAECLQRGVSPSQ 237
Tilapia     152 ALYTWYVKKQCEISQQFTNAKHGLATVKDQGEDT--KKGRRNRFKWGPASLQILFHAYERQKNPSKEEREGLVEECNRAECLQRGVSPSQ 239
Xenopus     159 ALYTWYVGKQRDIAKQFTHAGQGMLTDDMSCDEAPNKKMRRNRFKWGPASQQILFQAYERQKNPSKEEREALVEECNRAECLQRGVSPSQ 248
Chicken     164 ALYTWYVRKQREVAQQFTHAGQGILTEEPMGDDLPTKKGRRNRFKWGPASQQILFQAYERQKNPSKEEREALVEECNRAECIQRGVSPSQ 253
Alligator   164 ALYTWYVRKQREVAQQFTHAGQGILAEEPMGDDLPTKKGRRNRFKWGPASQQILFQAYERQKNPSKEEREALVEECNRAECIQRGVSPSQ 253
Mouse       161 ALYTWYVRKQREVAQQFTHAGQGGLIEEPTGDELPTKKGRRNRFKWGPASQQILFQAYERQKNPSKEERETLVEECNRAECIQRGVSPSQ 250
Human       161 ALYTWYVRKQREVAQQFTHAGQGGLIEEPTGDELPTKKGRRNRFKWGPASQQILFQAYERQKNPSKEERETLVEECNRAECIQRGVSPSQ 250

Grass carp  240 LAGLGSNLVTEVRVYNWFANRRKEEAFRHKLALDVPYSSQ-------------TASSTGQTLPSSPSPGLKYSQSVLCESLGTMRSSSG- 315
Zebrafish   238 LAGLGSNLVTEVRVYNWFANRRKEEAFRHKLALDVPYSSQ-------------SAASTCQTLPSSPSPGLKYSQTVVCESLGTVRSSGG- 313
Tilapia     240 LAGLGSNLVTEVRVYNWFANRRKEEAFRHKLALDTPFTSQ-------------TASSSNANLPPSPEHGVKYSQQIPCDTVSSARGSGG- 315
Xenopus     249 AQGLGSNLVTEVRVYNWFANRRKEEAFRHKLAMDTYNGQQ-----------SSAPPLSVHDLPHGKAPGLRYTQDSSTDR---------- 317
Chicken     254 AQGLGSNLVTEVRVYNWFANRRKEEAFRHKLAMDNFSGPQPTSAP-PLTPHNSSSLQPPPALSPTKVHGVRYNQQPASEAESSSSNHHGN 342
Alligator   254 AQGLGSNLVTEVRVYNWFANRRKEEAFRHKLAMDTYSTPQPNSAP-SLTSHSSSSLQPPPALSPSKVHGVRYSQQPVSEPESSSSNHHGN 342
Mouse       251 AQGLGSNLVTEVRVYNWFANRRKEEAFRHKLAMDTYNGPPPGPGPGPALPAHSSPGLPTTTLSPSKVHGVRYGQSATSEAAEVPSSSGG- 339
Human       251 AQGLGSNLVTEVRVYNWFANRRKEEAFRHKLAMDTYSGPPPGPGPGPALPAHSSPGLPPPALSPSKVHGVRYGQPATSETAEVPSSSGG- 339

                                  Transactivation domain
Grass carp  316 ---EGRAGSGRLSSPVQLEPSHTLLDTHHHKSVPGGGSLPPVSTLTSLHGVSGS----SAGPPGLIMASLPSVMSLG------------- 387
Zebrafish   314 ---EDRGASVRLASPVQLEPSHTLLETHHHKPASVGGSLPPVSTLTSLHGMSGS----SAGAPGLFIP---SVMSLG------------- 380
Tilapia     316 ---ERVG--RLMVSPVQLEPSHTLLETHNPKLVSSGGPLPPVSTLTSLHSLSAS----PASSQSLIMASVPSVMSLG------------- 383
Xenopus     318 ----SAAMVNSTLSPSALEPSNSLMNSDSKMVPISGGSLPPVSTLTALHSVDHSQHTLG-QTQNLIMASLPSVMTIG--TDTALGPAFSN 400
Chicken     343 SSMVTTQTILHQVSPPGLEPSQNLLSTDTKLISAPGGTLPPVSTLTALHSLEQNPHALGQQTQNLIMASLPGVMAIGAGETSSLAPAFTN 342
Alligator   343 NSMVTTQTILHQVSPPGLEPSQNLLSTDTKLISAPGGPLPPVSTLTALHNLEQNPHSLSQQTQNLIMASLPGVMAIGAGETSSLAPAFTN 432
Mouse       340 -PLVTVSAALHQVSPTGLEPS-SLLSTEAKLVSATGGPLPPVSTLTALHSLEQTSPGLNQQPQNLIMASLPGVMTIGPGEPASLGPTFTN 427
Human       340 -PLVTVSTPLHQVSPTGLEPSHSLLSTEAKLVSAAGGPLPPVSTLTALHSLEQTSPGLNQQPQNLIMASLPGVMTIGPGEPASLGPTFTN 428

Grass carp  386 --DSSLLIGLTSSQPQTVPVINNMGGGFTTLQPISFQQQLQASPQQPIAQQLQSHISPSSFMATMAQFP--CHMYS-KADLSSYPSSSLL 470
Zebrafish   381 --DSSLLIGLTSSQPQTVPIINNVGGGFTTLQPISFQQPLHASPQQPIAQQLQSHIAPSSFMATMAQLP--CHMYS-KADLSSYPSSSLL 465
Tilapia     384 --ESSLLIGLASTQPQTVPVINNMGGGFTTLQPISFQQQLHASPQPPIPQQLQSHMAASPFMATMAQLP--CHMYS-KSDSPQYHPSSLL 468
Xenopus     401 PGSSTLVIGLAS-QTQSVPVINSVGSSLTTLQSVQFSQQLHPSHQQPIVQQVQSHMAQSPFMATMAQLQPPHALYSHKPEVAQYASAGFF 489
Chicken     343 TGGSTLVIGLTSTQPQSVPVINSMGSSLTTLQPVQFSQQLHPSYQQPLMQQVQSHINQSPFMATMAQIQNPHALYGPKSEVAQYTHTGLL 522
Alligator   433 TGASTLVIGLASTQAQSVPVINSMGSSLTTLQPVQFSQQLHPSYQQPIMQQVQSHINQSPFMATMAQIQSPHALYGPKSEVAQYTHTGLL 522
Mouse       428 TGASTLVIGLASTQAQSVPVINSMGSSLTTLQPVQFSQPLHPSYQQPLMPPVQSHVAQSPFMATMAQLQSPHALYSHKPEVAQYTHTSLL 517
Human       429 TGASTLVIGLASTQAQSVPVINSMGSSLTTLQPVQFSQPLHPSYQQPLMPPVQSHVTQSPFMATMAQLQSPHALYSHKPEVAQYTHTGLL 518

Grass carp  471 SQAMVIADSNSIGTLTNLTAVRQILTSDPEGHTDT------------PIEEES----LHLQS----TSPEPASSGSLELYPQTQTSESHP 540
Zebrafish   466 SQAMVIADSSSIGTLTNLTAVRQILTTDPEGHTES------------AIEEDS----LHLQS----TSPEAGSSGSLDLYPQSQTSETHS 535
Tilapia     469 SQAMVIADSS---SLASLTAVRQILTTDPDEQEDA------------PLQEDS----LNMQP----HSPAPVSSESLELYPSSQATESHQ 535
Xenopus     490 PQTMVITDTSNLGTLTSLTPSKQVVPLHPIAQGDS----PGS-----HLQDSS---ILHLHHSHRLSPIPTVSSASLAHYQNSSSPENHS 567
Chicken     523 PQTMVITDTANLSALTNLTPTKQAFTSDSETHTDSGIHTPVSQAPAIHLQNQD-TAIQHLQSGPRLTPSPAVSSSSLVLYQSSDSTNSHS 611
Alligator   523 PQTMLITDTANLSALTNLTPTKQVFTSDSENHTESGIHTPVSQTQTIHLQNQD-TTIQHLQPGPRLTSSPAASSSSLVLYQSSDSTNSHS 611
Mouse       518 PQTMLITDT-NLSTLASLTPTKQVFTSDTEASSEPGLHEPPSPATTIHIPSQDPSNIQHLQPAHRLSTSPTVSSSSLVLYQSSDS-NGHS 605
Human       519 PQTMLITDTTNLSALASLTPTKQVFTSDTEASSESGLHTPASQATTLHVPSQDPAGIQHLQPAHRLSASPTVSSSSLVLYQSSDSSNGQS 608

                                            Identity (%)
Grass carp  541 SHLLSSSP-GDIDPYIPTQMVSTAQ 564 100.0
Zebrafish   536 SHLQLSSPAGDIDPYIPAQMVSTAQ 560  87.8
Tilapia     536 SHLLSPSP-TEISSYIPAQMVSTAQ 559  73.6
Xenopus     568 HLLSPSHS--TIDSFMSTQMASSSQ 590  48.7
Chicken     612 QLLPSTHN--VIETFISTQMASSTQ 634  47.8
Alligator   612 HLLPSTHS--VIETFISAQMASSTQ 634  47.7
Mouse       606 HLLPSNHS--VIETFISTQMASSSQ 628  47.0
Human       609 HLLPSNHS--VIETFISTQMASSSQ 631  46.5
```

Supplemental Fig.2. Protein sequence alignment of carp HNF1α with the corresponding sequences in other species. Sequence alignment was conducted using Clustal-W algorithm. The conserved residues in the protein sequences used for comparison are shaded in grey for recognition. The 4 structural domains for HNF1α, including the dimerization domain, POU domain, homeobox domain and transactivation domain, are also delineated by horizontal lines on the top of the respective region for the HNF1α sequences presented.
