# Supplementary material for: Novel Mechanisms for IGF-I Regulation by Glucagon in Carp Hepatocytes: Up-Regulation of HNF1α and CREB Expression via Signaling Crosstalk for IGF-I Gene Transcription
Source: Front Endocrinol (Lausanne). 2019 Sep 3;10:605. doi: 10.3389/fendo.2019.00605 (PMC6734168; doi:10.3389/fendo.2019.00605)
Supplement: Supplementary file 4 [file Data_Sheet_4.PDF]

Supplemental Fig.3

A

Grass Carp HNF1a (Protein coverage by peptides listed in B: 57.3%, grass carp liver)

```
MEGGEGRRAGGERSSRLSALQEQLVWSLLGSGLSKELLIQAMGDLERERASTGAERTDRADGESSEEGEMENPPPIFHDLERLPPEEAARQRAEV
  Peptide 1
DQLLQEDPWHVAKIVKSYMQQHNLPQREVVESTGLNQSHLSQHLNKGTPMKNQKRAALYSWYIKKQTEISQQFTNASRGVMSGEESGEDVRKGRR

NRFKWGPASQQILFQAYERQKNPSKEEREGLVEECNRAECLQRGVSPSQLAGLGSNLVTEVRVYNWFANRRKEEAFRHKLALDVPYSSQTASSTG
                  Peptide 2
QTLPSSPSPGLKYSQSVLCESLGTMRSSSGEGRAGSGRLSSPVQLEPSHTLLDTHHHKSVPGGGSLPPVSTLTSLHGVSGSSAGPPGLIMASLPS
                          Peptide 3
VMSLGDSSLLIGLTSSQPQTVPVINNMGGGFTTLQPISFQQQLQASPQQPIAQQLQSHISPSSFMATMAQFPCHMYSKADLSSYPSSSLLSQAMV

IADSNSIGTLTNLTAVRQILTSDPEGHTDTPIEEESLHLQSTSPEPASSGSLELYPQTQTSESHPSHLLSSSPGDIDPYIPTQMVSTAQ
```

B

Peptides identified with 99% confidence

Conf.	Peptide Sequence	ΔMass	Prec MW	z	Prec m/z
99	AALYSWYIK	0.0080	1722.01	4	431.51
99	ADLSSYPSSSLL	0.0390	1542.85	3	515.29
99	ADSNSIGTLTN	0.0580	1395.77	2	698.89
99	AECLQR	0.0360	1069.56	2	535.79
99	AECLQRGVSPSQLAGLGSNLVTEVR	0.1980	2933.74	4	734.44
99	AEVDQLLQEDPWHVAK	0.0440	2485.39	4	622.35
99	AGGERSSR	0.0070	1122.61	2	562.31
99	AGLGSNLVT	-0.0100	1134.65	2	568.33
99	ASTGAER	0.0480	994.58	2	498.30
99	ASTGAERTDR	0.1130	1366.82	3	456.62
99	ASTGAERTDRA	0.1530	1494.92	3	499.31
99	EEAFR	0.0280	954.54	2	478.28
99	EEAFRHK	-0.0350	1523.83	3	508.95
99	EGGEGRR	0.0470	1063.62	2	532.81
99	EGGEGRRAGGER	0.0420	1533.83	2	767.92
99	ELLIQAMGDLER	-0.0510	1706.87	2	854.44
99	ELLIQAMGDLERERASTGAER	0.0070	2704.46	3	902.49
99	ELLIQAMGDLERERASTGAERTDR	0.1210	3020.68	3	1007.90
99	ERASTGAER	0.0100	1279.69	2	640.85
99	GEGRR	0.0680	877.57	2	439.79
99	GEGRRAGGER	0.1430	1347.87	3	450.30
99	GEGRRAGGERSSR	0.0760	1677.97	3	560.33
99	GGEGRR	0.0390	934.57	2	468.29
99	GGEGRRAGGER	0.1500	1404.90	3	469.31
99	GGEGRRAGGERSSR	0.2570	1735.17	4	434.80
99	GGEGRRAGGERSSRL	0.0170	1848.02	3	617.01
99	GTMRSSSGEGR	0.0970	1139.63	2	570.82
99	GTPMK	0.0140	1140.69	2	571.35
99	GVSPSQLAGLGSNLVTEVR	-0.0480	2244.20	2	1123.11
99	ISPSSF	-0.0020	940.52	2	471.27
99	IVKSY	0.0210	1216.79	2	609.40
99	LALDVPYSSQTASSTGQTLPSSPSPGLK	0.0340	3396.86	5	680.38
99	LIQAMGDLERERASTGAER	-0.0300	2390.27	3	797.76
99	LPPEEAAR	-0.0890	1185.58	2	593.80
99	LPPEEAARQR	0.0080	1470.82	3	491.28
99	MEGGEGR	0.0970	1038.60	2	520.31
99	MEGGEGRR	0.0330	1194.64	2	598.33
99	MEGGEGRRAGGER	0.0940	1664.93	2	833.47
99	NPSK	-0.0130	1053.62	2	527.81
99	NPSKEER	-0.0560	1467.76	2	734.89
99	NQKR	-0.0710	1153.63	2	577.82
99	PSSPSPGLK	0.0420	1476.92	3	493.31
99	QAMGDLER	0.0260	1165.63	2	583.82
99	QKNPSK	0.0360	1613.04	3	538.69
99	QRAEVDQLLQEDPWHVAK	-0.0260	2770.46	4	693.62
99	QTEISQQFTNASR	0.1650	1813.09	3	605.37
99	RAALYSWYIK	-0.0610	1878.04	3	627.02
99	RAGGER	0.0720	948.61	2	475.31
99	RAGGERSSR	0.0300	1278.74	3	427.25
99	SALQEQLVWSLLGSGLSK	0.0340	2523.49	3	842.17
99	SSSGEGR	0.0640	982.56	2	492.29
99	SSSGEGRAGSGR	0.0450	1410.76	2	706.39
99	SVPGGGSL	0.0530	976.60	2	489.31
99	SYMQQHNLPQR	0.1530	1705.02	4	427.26
99	TTLQPISFQ	-0.0220	1337.73	2	669.87
99	VYNWFANRRK	0.2660	1961.39	4	491.35
99	WGPASQQILFQAYER	0.1780	2097.27	3	700.10

C

Representative mass spectra

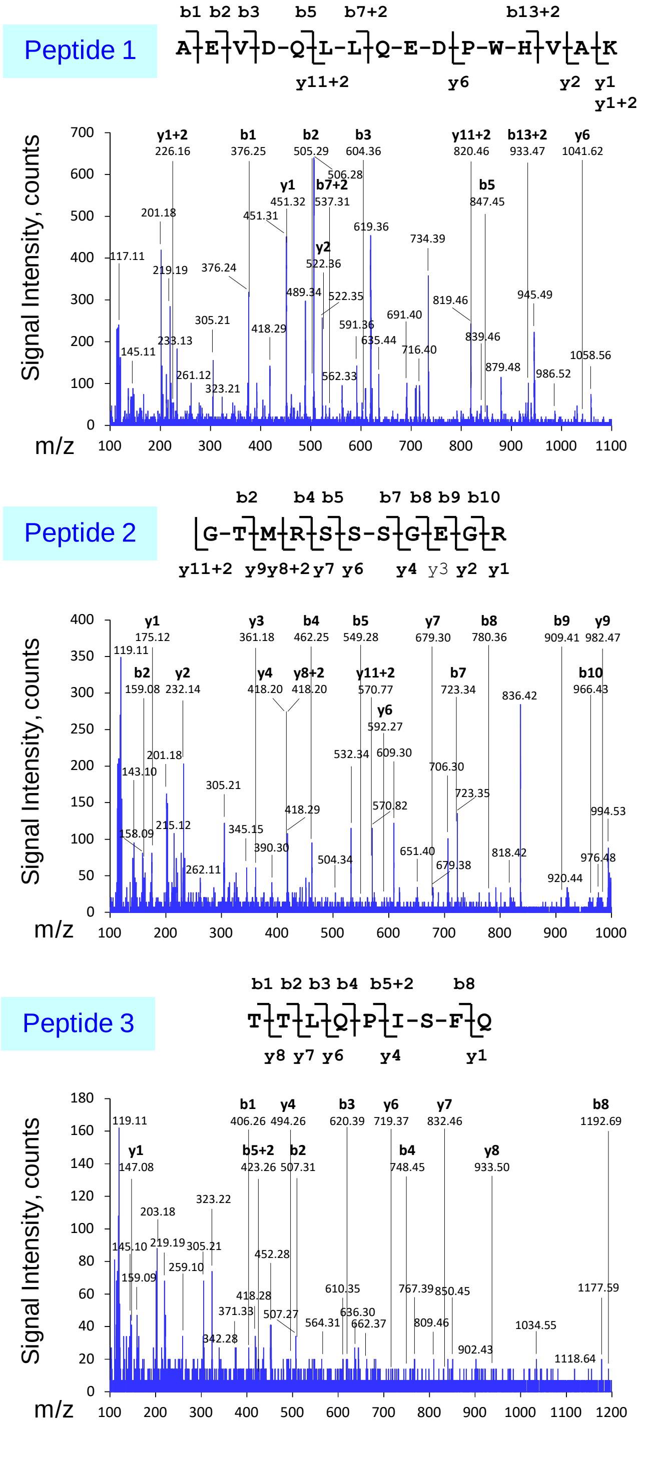

Supplemental Fig.3. Detection of protein expression of HNF1α in the carp liver with the proteomic approach. Protein lysate was prepared from the carp liver and subjected to trypsin digestion followed by LC/MS/MS. The digested products were then resolved by C_{18} chromatography followed by MS/MS detection. Peptide products originated from HNF1α were identified by ProteinPilot 2.01 based on the a.a. sequence of carp HNF1α presented in Supplemental Fig.1. For MS/MS data presented, locations of the peptides identified with confidence level at 99% were mapped (by underlines) in the full-length a.a. sequence of carp HNF1α presented in (A). The sequences of the peptides identified together with the corresponding QC data, including the percentage of confidence (as "Confid.") and mass derivation (as "ΔMass"), and MS data, including precise molecular weight (as "Prec MW"), theoretical charge (as "Z") and precise mass-to-charge ratio (as "Prec m/z"), are presented in (B). Representative MS/MS spectra of peptides originated from HNF1α, including peptide 1, 2 and 3 (shaded in grey within the a.a. sequence of carp HNF1α), are presented in (C). For MS/MS spectra, the ion peaks corresponding to the series of b- and y-fragments generated by collision-induced fragmentation have been annotated for the respective peptide targets.
